# Single‐Cell Landscape Highlights Heterogenous Microenvironment, Novel Immune Reaction Patterns, Potential Biomarkers and Unique Therapeutic Strategies of Cervical Squamous Carcinoma, Human Papillomavirus‐Associated (HPVA) and Non‐HPVA Adenocarcinoma

**DOI:** 10.1002/advs.202204951

**Published:** 2023-02-01

**Authors:** Junjun Qiu, Xinyu Qu, Yumeng Wang, Chenyan Guo, Bin Lv, Qian Jiang, Wentao Su, Li Wang, Keqin Hua

**Affiliations:** ^1^ Department of Gynecology Obstetrics and Gynecology Hospital Fudan University 419 Fangxie Road Shanghai 200011 China; ^2^ Shanghai Key Laboratory of Female Reproductive Endocrine‐Related Diseases 413 Zhaozhou Road Shanghai 200011 China; ^3^ School of Food Science and Technology Dalian Polytechnic University Dalian 116034 China; ^4^ Institutes of Biomedical Sciences Fudan University No. 130 Dongan Road Shanghai 200032 China; ^5^ Center for Medical Research and Innovation Shanghai Pudong Hospital Fudan University Pudong Medical Center 2800 Gongwei Road, Pudong Shanghai 201399 China

**Keywords:** cervical cancer, HPV infection, precise treatment, scRNA‐seq, TCR‐seq, transcriptional heterogeneity, tumor microenvironment

## Abstract

Cervical adenocarcinomas (ADCs), including human papillomavirus (HPV)‐associated (HPVA) and non‐HPVA (NHPVA), though exhibiting a more malignant phenotype and poorer prognosis, are treated identically to squamous cell carcinoma (SCC). This clinical dilemma requires a deeper investigation into their differences. Herein a transcriptomic atlas of SCC, HPVA, and NHPVA‐ADC using single‐cell RNA (scRNA) and T‐cell receptor sequencing (TCR‐seq) is presented. Regarding structural cells, the malignancy origin of epithelial cells, angiogenic tip cells and two subtypes of fibroblasts is revealed. The promalignant properties of the structural cells using organoids are further confirmed. Regarding immune cells, myeloid cells with multiple functions other than antigen presentation and exhausted T lymphocytes contribute to immunosuppression. From the perspective of HPV infection, not only is HPV‐dependent and independent cervical cancer oncogenesis proposed but also three immune reaction patterns mediated by T cells (coordinated/inactive/imbalanced) are identified. Strikingly, diagnostic biomarkers to distinguish ADC from SCC are discovered and prognostic biomarkers with marker genes for malignant epithelial cells, tip cells, and SPP1/C1QC macrophages are generated. Importantly, the efficacy of anti‐CD96 and anti‐TIGIT, not inferior to anti‐PD1, in animal experiments is confirmed and targeted therapies specifically for HPV‐positive SCC, HPVA and NHPVA‐ADC, providing essential clues for further clinical trials, are proposed.

## Graphical abstract

A comprehensive single‐cell atlas of cervical cancer (CC), including HPV‐positive squamous cell carcinoma (SCC), HPV associated (HPVA) and non‐HPV associated (NHPVA) adenocarcinoma, is depicted. Through verification from organoid culture, mice experiments and clinical samples, the complex CC microenvironment is unveiled, novel clinical biomarkers are generated, and promising precise treatment modalities are proposed, providing essential clues for further CC clinical trials.

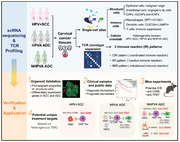

## 1 Introduction

Cervical cancer (CC) remains one of the leading causes of death among women worldwide.^[^ ^1^ ^]^ The most common subtypes of CC are squamous cell carcinoma (SCC) and adenocarcinoma of the cervix (ADC), comprising up to 70% and 25% of all cases, respectively.^[^ ^2^, ^3^ ^]^ Compared with SCC, ADC manifests a worse prognosis owing to its lack of symptoms on clinical examination and greater propensity for metastasis, recurrence, and resistance to chemotherapy.^[^ ^4^, ^5^, ^6^ ^]^ However, patients suffering from ADC and SCC receive the same conventional treatment strategies. Therefore, it is of vital importance to elucidate the differences between SCC and ADC, especially the unique molecular mechanism of ADC considering its more malignant phenotype and poorer prognosis, which will hopefully provide new insights for precision treatment.

In 2018, ADC was categorized into human papillomavirus (HPV)‐associated (HPVA) and non‐HPV‐associated (NHPVA) based on both morphology and etiology using the International Endocervical Adenocarcinoma Criteria and Classification, which is more reproducible and clinically informative than the 2014 WHO classification based on only descriptive morphological features.^[^ ^7^, ^8^ ^]^ Since the adoption of this novel pathology scheme in the National Comprehensive Cancer Network guidelines in 2020,^[^ ^9^ ^]^ interest in ADC research has dramatically increased. In fact, high‐risk HPV gene integration into the host gene^[^ ^10^ ^]^ was found to be responsible for the oncogenesis of HPVA. However, the pathological mechanisms underlying NHPVA remain unknown. Although previous studies have illustrated gene mutations and pathway activation profiles among ADC cases,^[^ ^11^, ^12^ ^]^ the differences between HPVA and NHPVA have not been well established. Additionally, studies applying genomic and transcriptome sequencing to ADC are limited to the analysis of bulk cells, reflecting the average profiles of tumor samples with low resolution. Therefore, detailed insight into the cellular composition, function, and microenvironment within the tumor is urgently needed to decipher the differences between HPVA and NHPVA ADCs.

Recently, single‐cell RNA sequencing (scRNA‐seq), emerging as a cutting‐edge technology, has yielded unprecedented insights into intra‐tumoral and inter‐tumoral heterogeneities,^[^ ^13^ ^]^ and has redefined our understanding of tumor oncogenesis, recurrence, metastasis and therapy resistance.^[^ ^14^, ^15^ ^]^ In the field of CC, studies utilizing scRNA‐seq dealt with the heterogeneity of cervical cancer in different clinical stages with a small sample size,^[^ ^16^ ^]^ limiting the breadth and depth of the research. The complexity of the tumor microenvironment (TME), differences between different histologic subtypes and the association between HPV infection and tumorigenesis have not been systemically studied.

The tumor immune microenvironment (TIME) is not only a cause of tumors whose dysregulation facilitates immune escape and tumor initiation but also a unique consequence, displaying a complicated and immunosuppressive phenotype as the tumor advances.^[^ ^17^ ^]^ Studies on the TIME of CC and clinical trials of immune checkpoint blockades (ICBs), including anti‐CTLA4 and anti‐PD1 agents, have been extensively conducted and have dramatically altered the landscape of cancer treatment.^[^ ^18^ ^]^ However, in CC, the response rate to immunotherapies is far from satisfactory.^[^ ^19^ ^]^ Therefore, it is paramount to unravel the mechanisms and pathways underlying this low efficacy and discover better strategies to direct the immune system to fight CC. Fortunately, T‐cell receptor sequencing (TCR‐seq) allows the recognition of viral antigens and tumor‐specific neoantigens and illustrates clonal expansion patterns as well as differentiation lineages of T cells.^[^ ^20^ ^]^ Hence, combining scRNA‐seq with TCR‐seq may help to uncover the unique TIME of CC.

In the current study, we performed scRNA‐seq and TCR‐seq with SCC, HPVA and NHPVA ADC samples, depicting a comprehensive single‐cell atlas of CC. Remarkably, by combining high‐throughput sequencing analyses with verification from 3D organoid culture, mouse experiments, clinical samples and The Cancer Genome Atlas (TCGA) public data, we not only unveil the complex TME of CC from the perspective of structural cells, immune cells and cellular crosstalk, highlight the mechanisms of HPV remodeling in the CC TME but also generate novel diagnostic and prognostic biomarkers for CC as well as promising precise treatment modalities specifically for HPV‐positive SCC, HPVA ADC and NHPVA ADC. Most importantly, we propose that anti‐CD96 and anti‐TIGIT would not be inferior to anti‐PD1, offering some basic evidence for further clinical investigation.

## 2 Results

### 2.1 Single‐Cell Transcriptome Atlas of SCC and ADC

**Figure 1:**
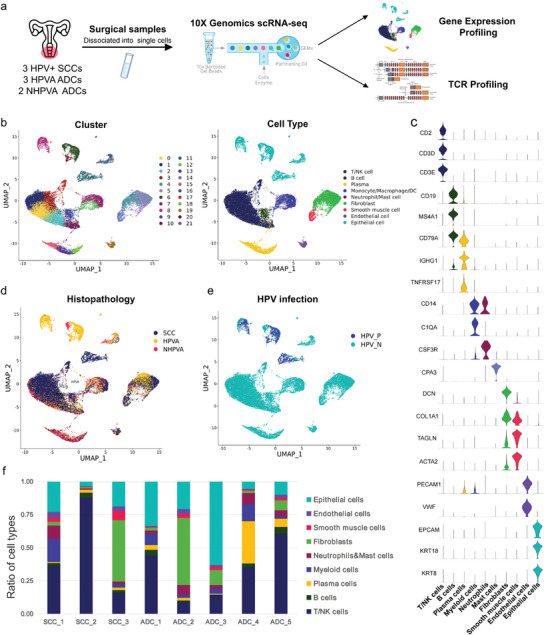
Single‐cell transcriptome atlas of SCC and ADC. A) Schematic diagram of conducting scRNA‐seq and TCR‐seq of eight CC samples including three HPV‐positive SCC samples, three HPVA ADC samples, and two NHPVA ADC samples through the 10× Genomics Platform. B) UMAP projection of 29 568 cells which were clustered into 22 clusters (left) and further categorized into nine major cell types (right). Each single dot corresponds to one single cell colored according to cell cluster (left) or cell type (right). C) Violin plots showing the expression of well‐recognized marker genes in the major cell types. D) UMAP projection of all the cells presenting in different histologic subtypes. Each single dot corresponded to one single cell colored according to histologic subtypes including SCC, HPVA, and NHPVA ADC. Epithelial cells are aggregated merely within a small part between SCC and ADC (cluster 16), whereas other cell types showed adequate integration. E) UMAP projection of all the cells presenting with HPV infection status. Each single dot corresponds to one single cell colored according to HPV infection status. HPV only infects the epithelial cells in the CC TME, thus mediating the oncogenesis. F) Histogram indicating the ratios of each major cell type in eight CC samples, respectively, indicating remarkable inter‐tumoral heterogeneity. SCC, squamous cell carcinoma; ADC, cervical adenocarcinoma; scRNA, single‐cell RNA; TCR‐seq, T‐cell receptor sequencing; CC, cervical cancer; HPV, human papillomavirus; NHPVA, non‐human papillomavirus‐associated; TME, tumor microenvironment.

To decipher the TME of SCC and ADC, we conducted scRNA‐seq and TCR‐seq on three HPV‐positive SCC samples, three HPVA ADC samples and two NHPVA ADC samples (**Figure** **1**A and Table S1, Supporting Information). After standard data processing and quality control procedures, 29 568 cells were subsequently reclustered into 22 clusters through principal component analysis (PCA) and partitioned into nine major groups (Figure 1B), annotated by known cell‐type marker genes^[^ ^14^, ^21^ ^]^ (Figure 1C): T cells (*CD2*, *CD3D*, and *CD3E*), B cells (*CD19*, *CD79A*, and *MS4A1*), plasma cells (*IGHG1* and *TNFRSF17*), myeloid cells including monocytes, macrophages and dendritic cells (*CD14* and *C1QA*), neutrophils/mast cells (*CSF3R*/*CPA3*), fibroblasts (*DCN* and *COL1A1*), smooth muscle cells (*TAGLN* and *ACTA2*), endothelial cells (*PECAM1* and *VWF*) and epithelial cells (*EPCAM*, *KRT18*, and *KRT8*). The proportion of each cell type varied greatly in different cervical cancer tissues (Figure 1F), demonstrating inter‐tumoral heterogeneity. Notably, the epithelial cells were mostly histology‐specific, whereas other types of cells including endothelial cells, fibroblasts and immune cells from SCC, HPVA and NHPVA ADC samples aggregated well (Figure 1D), indicating the uniqueness of epithelial cells but the commonness of other cells in the microenvironment of cervical cancer with different histologic subtypes. In addition, HPV infection only occurred in epithelial cells among all cell types, potentially revealing the origin of the cervical malignant transition (Figure 1E).

Since epithelial cells, fibroblasts and endothelial cells are structural cells^[^ ^22^ ^]^ and are major components of the TME, we next focused on these three cell types and explored their heterogeneity in the CC TME.

### 2.2 Structural Cells: Indispensable Components in the CC TME

#### 2.2.1 Heterogenous Epithelial Cells: Mediating Tumorigenesis Respectively in SCC and ADC

**Figure 2:**
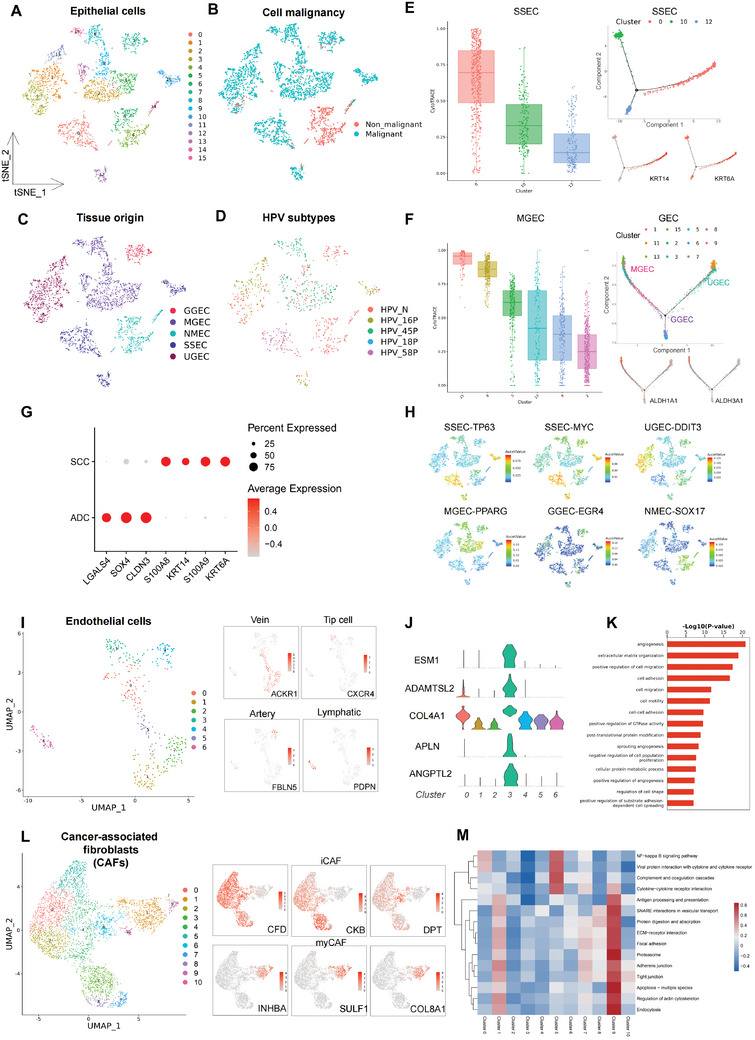
Epithelial cells turning malignant, tip cells mediating angiogenesis and fibroblasts acting as accomplices in CC TME. A) tSNE projection of the 16 epithelial subclusters generated from unsupervised reclustering. See also Figure S1A, Supporting Information. B) tSNE projection of malignant (subclusters 0, 1, 2, 3, 5, 7, 8, 9, 10, 11, 12, 13, and 15) and nonmalignant (subclusters 4, 6, and 14) epithelial cells determined by inferCNV analysis. See also Figure S1B,C, Supporting Information. C) tSNE projection of five major types of epithelial cells indicating tissue origin. See also Figure S1F, Supporting Information. D) tSNE projection of epithelial cells with HPV infection statuses. See also Figure S1G, Supporting Information. E) Predicted differentiation order of SSECs by CytoTRACE analysis with higher CytoTRACE score representing less differentiation extent and greater developmental diversity (left) and trajectory of differentiation of SSECs predicted by monocle (right) with the expression of *KRT14* and *KRT6A* (markers for basal epithelial cells). F) Predicted differentiation order of MGECs by CytoTRACE analysis (left) and trajectory of differentiation of GECs predicted by monocle (right) with the expression of *ALDH1A1* and *ALDH3A1* (markers for stem cells). G) Dot plot indicating the average expression levels and cell expressing proportion of differentially expressed genes (DEGs) between SSECs and GECs. The colors represent the average expression levels, and dot sizes represent the percentage expression of selected genes. H) tSNE projection of specific transcription factors (TFs) regulating the five major types of CC epithelial cells (*TP63* and *MYC* mainly regulating SSECs; *DDIT3* mainly regulating UGECs; *PPARG* mainly regulating MGECs; *EGR4* mainly regulating GGECs; and *SOX17* mainly regulating NMECs). Color key from blue to red indicates AUCell value from low to high. I) UMAP projection of the seven subclusters generated from endothelial cell reclustering (left) and the expression levels of the specific marker gene for the four major types of endothelial cells (right): *ACKR1* for vein cells (subclusters 0, 1, 2, and 5); *CXCR4* for tip cells (subcluster 3); *FBLN5* for artery cells (subcluster 4); and *PDPN* for lymphatic cells (subcluster 6). Color key from gray to red indicates relative expression levels from low to high. J) Violin plots indicating the expression levels of the marker genes for tip cells (*ESM1*, *CLO4A1*, *ADAMTSL2*, *APLN*, and *ANGPT2*). K) Bar chart showing the enrichment of angiogenesis‐related pathways based on the GO pathways in tip cells presented with statistical significance [−Log10(P‐value)]. L) UMAP projection of the 11 subclusters generated from CAFs reclustering (left) and the expression levels of the specific marker genes for the two major types of CAFs (right): *CFD*, *CKB*, and *DPT* for iCAFs (subclusters 0, 2, 3, 4, 5, 6, 7, and 8); *INHBA, SULF1, COL8A1* for myCAFs (subclusters 1, 9, and 10). Color key from gray to red indicates relative expression levels from low to high. M) Heatmap demonstrating the KEGG pathway QuSAGE enrichment scores for each CAF subcluster. A score of 0 (white color) represents non‐significant enrichment after FDR correction. CC, cervical cancer; TME, tumor microenvironment; SSECs, stratified squamous epithelial cells; CytoTRACE, Cellular Trajectory Reconstruction Analysis using gene Counts and Expression; MGECs, mucinous glandular epithelial cells; GECs, glandular epithelial cells; DEGs, differentially expressed genes; TFs, transcription factors; GO, gene ontology; CAFs, cancer‐associated fibroblasts; iCAFs, inflammatory cancer‐associated fibroblasts; myCAFs, myoblastic cancer‐associated fibroblasts; KEGG, Kyoto Encyclopedia of Genes and Genomes; FDR, false discovery rate.

We obtained 16 subclusters of epithelial cells through unsupervised reclustering (**Figure** **2**A and Figure S1A, Supporting Information). By inferCNV analysis (Figure S1B, Supporting Information), we successfully identified nonmalignant and malignant cells (Figure 2B). The analyses of differentially expressed genes (DEGs) between malignant and nonmalignant epithelial cells (NMECs), as well as subsequent enriched functional pathways (Figure S1C–E, Supporting Information), indicated that nonmalignant cells might play a unique role in antiviral reactions, while malignant cells are closely related to epithelial cell differentiation and keratinization.

To clarify the origin of epithelial cells, we categorized the aforementioned 16 subclusters into the following five types based on gene expression^[^ ^23^, ^24^ ^]^ (Figure 2C and Figure S1F, Supporting Information): 1) stratified squamous epithelial cells (SSECs; *KRT5*); 2) gastrointestinal glandular epithelial cells (GGECs; *ANXA10*, *CLDN18*); 3) mucinous glandular epithelial cells (MGECs; *MUC17* and *CLDN15*); 4) usual‐type glandular epithelial cells (UGECs; *MUC16*); and 5) NMECs (*SOX17*). To elucidate the relationship between HPV infection and cell malignancy, we compared the distribution of HPV‐infected epithelial cells (Figure 2D) and inferred malignant cells, which were consistent with each other. Indeed, HPV infection plays a significant role in CC oncogenesis, characterized by high expression of *CDKN2A*, *CLDN3*, *CLDN4* and *GDF15* (Figure S1G, Supporting Information), influencing proliferative signaling, cell cycle and tight junctions. Furthermore, we noticed that some HPV‐negative cells, such as GGECs in subcluster 7, were inferred to be malignant and specifically expressed *ANXA10*, *CXCL5* and *CLDN18*, whose dysregulation influences signal transduction and interferes with cellular polarity, morphology and growth, thus leading to an unexpected differentiation path of cervical glandular epithelial cells (GECs) to the gastric‐type. These findings suggested a non‐HPV‐related pathogenic mode of CC malignancy. Together, these results demonstrated that the pathogenic mechanisms of HPV‐ and non‐HPV‐related CC remain fundamentally different.

Quantitative set analysis of gene expression (QuSAGE) was conducted to determine the biological functions of the aforementioned five types of epithelial cells (Figure S1H, Supporting Information). SSECs activated the MYC signaling pathway and interferon response. UGECs were positively associated with multiple common carcinogenesis pathways (epithelial–mesenchymal transition, hypoxia, and tumor necrosis factor signaling). Antigen processing and presentation were dramatically enriched in GGECs, whereas mismatch repair and peroxisome pathways were enriched in MGECs. In contrast, NMECs showed negative correlations with cancer‐related pathways (cell cycle, DNA replication, and p53 signaling), suggesting their unique anti‐tumor roles in CC. Moreover, we determined the potential developmental and differentiation trajectories of SSECs and GECs through Cellular Trajectory Reconstruction Analysis using gene Counts and Expression (CytoTRACE) and pseudotime analyses (Figure 2E,F). Among SSECs, basal epithelial cells (subcluster 0) featuring high expression of *KRT14* and *KRT6A*, exhibited stemness, differentiating into more mature cells (subclusters 10 and 12). Among MGECs, subclusters 15 and 8 expressed high levels of stem cell markers, such as *ALDH3A1* and *ALDH1A1*, highlighting their developmental diversity. Moreover, MGECs and GGECs were inferred to be the predecessors of UGECs; the malignancy of mucinous‐ and gastric‐type ADCs may be due to their high developmental diversity and poor differentiation compared with usual‐type ADCs. In summary, we discovered that cells with stemness and poor differentiation might be the origin of CC malignancy.

To further explore the molecular heterogeneity of SCC and ADC, we analyzed the DEGs and transcription factors (TFs) of the two histologic subtypes. Genes, such as *S100A8* and *KRT14* could serve as markers of SCC, while *LGALS4* and *CLDN3* were specifically expressed in ADC (Figure 2G). In addition, various types of epithelial cells were regulated by specific TFs (Figure 2H). Notably, *MYC* and *TP63* were dominant in regulating SSEC. In terms of GECs, UGECs, GGECs and MGECs were modulated mainly by *DDIT3* (associated with DNA binding and endoplasmic reticulum stress), *EGR4* (related to mitogenesis and differentiation), and *PPARG* (involved in epithelial cell differentiation), respectively. Additionally, *SOX17* (a marker for reserve cells) was found to regulate NMECs. Collectively, the DEGs and TFs of SCC and ADC hold promising potential to serve as diagnostic biomarkers.

#### 2.2.2 Tip Cells: Dominating Sprouting Angiogenesis

To better understand the features and functions of endothelial cells in CC TME, we reclustered endothelial cells and categorized them into 4 major groups^[^ ^25^ ^]^ (Figure 2I): 1) vein cells (*ACKR1*); 2) tip cells (*CXCR4*); 3) artery cells (*FBLN5*); and 4) lymphatic cells (*PDPN*). Remarkably, tip cells, a group of specialized endothelial cells with protruding filopodia, which guide vessel‐sprouting, attracted our attention. High expression of genes such as *ESM1*, *COL4A1*, *ADAMTSL2*, *ANGPTL2*, and *APLN* was observed in tip cells (Figure 2J). Additionally, gene ontology (GO) analysis confirmed the leading roles of tip cells in sprouting angiogenesis (Figure 2K). Thus, tip cells in the TME of CC play crucial roles in tumor angiogenesis and may serve as promising anti‐angiogenic targets.

#### 2.2.3 Two Subtypes of Cancer‐Associated Fibroblasts (CAFs): Acting as Accomplices in CC

Given that CAFs are reported to be an important stromal component and are critically involved in tumor progression,^[^ ^26^ ^]^ we reclustered the CAFs and further categorized them into two main types (Figure 2L): inflammatory CAFs (iCAFs; *CFD*, *CKB*, and *DPT*) and myoblastic CAFs (myCAFs; *INHBA*, *SULF1*, and *COL8A1*). QuSAGE functional analysis (Figure 2M) showed that iCAFs actively participated in the NFKB signaling pathway, complement and coagulation cascades, and cytokine–cytokine receptor interactions (Figure S2A, Supporting Information), emphasizing the importance of inflammation‐regulated carcinogenesis in the development of CC. In contrast, myCAFs played significant roles not only in extracellular matrix (ECM) remodeling (Figure S2B, Supporting Information) but also in antigen processing and presentation. Furthermore, although iCAFs and myCAFs possessed distinct expression patterns of growth factors (Figure S2C, Supporting Information), they acted together as accomplices in CC progression.

#### 2.2.4 Validation of Features and Function of Structural Cells in CC: Integrating scRNA‐seq with 3D Organoid Culture

**Figure 3:**
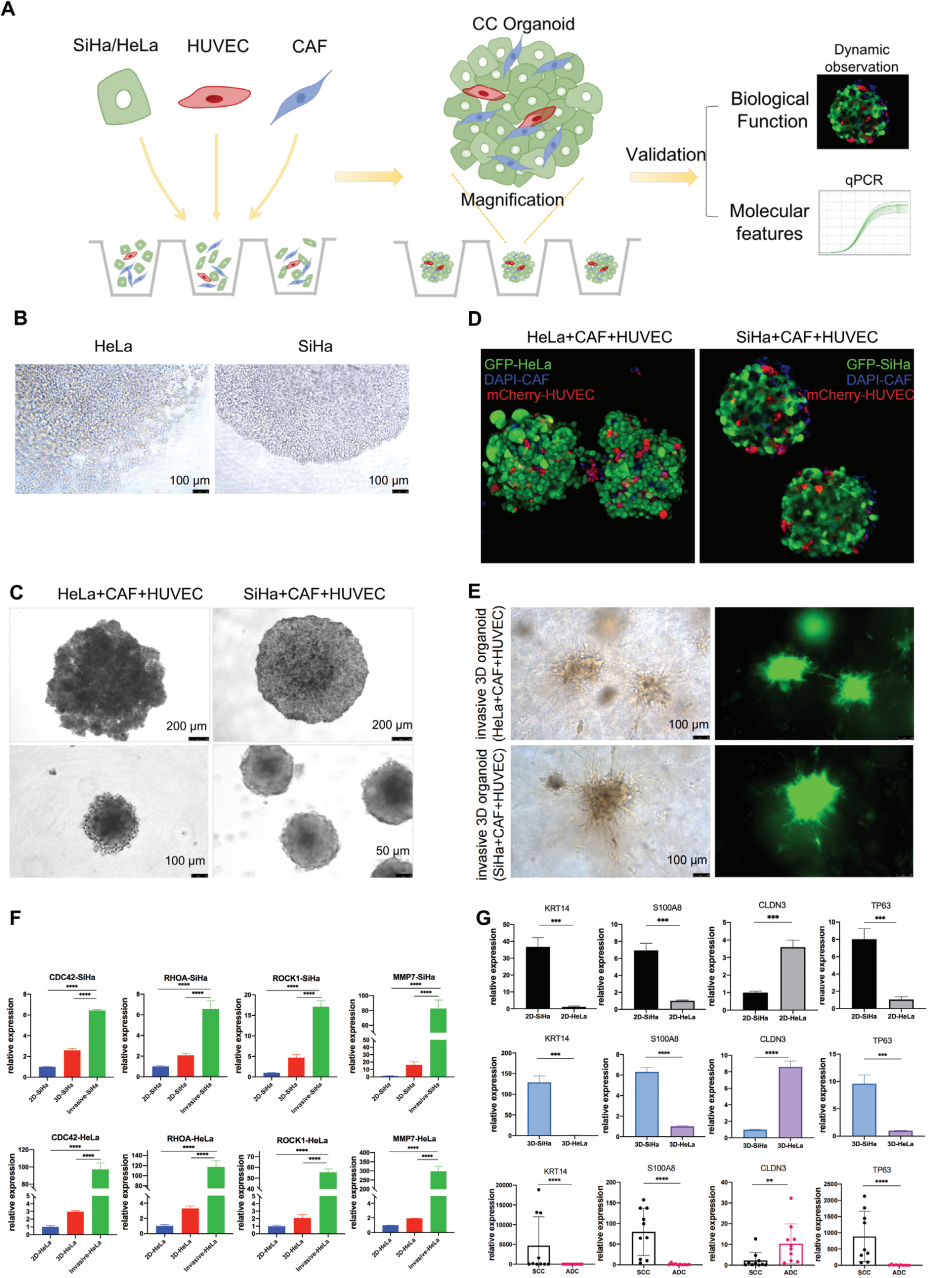
Validation of both the features and functions of structural cells in CC: integrating scRNA‐seq with 3D organoid culture. A) Scheme of establishing 3D organoids with SiHa/HeLa cell lines, CAFs, and endothelial cells to validate the features and functions of structural cells in CC identified by scRNA‐seq. B) 3D cultivation of SiHa or HeLa cells alone could not form spheroids (scale bars, 100 µm). C) ADC organoids consisting of HeLa cells, CAFs and HUVECs (scale bars, 200 [upper] and 50 µm [lower]) and SCC organoids consisting of SiHa cells, CAFs and HUVECs (scale bars, 200 [upper] and 100 µm [lower]) in bright fields. D) SCC and ADC organoids consisting of GFP‐SiHa/HeLa cells, mCherry‐HUVECs, and CAFs stained with DAPI observed by confocal microscopy (200× magnification). E) SCC and ADC organoids protruding into the Matrix gel exhibiting tumor invasiveness in bright field (left) and under fluorescence (right). Scale bars, 100 µm. F) qRT–PCR results indicating that *CDC42*, *RHOA*, *ROCK1*, and *MMP7* were more intensely expressed in invasive 3D organoids (invasive‐SiHa/invasive HeLa) than in static organoids (3D‐SiHa/3D‐HeLa) or SiHa/HeLa cells cultured in traditional 2D conditions (2D‐SiHa/2D‐HeLa). Error bar: mean value ± SD (*n* = 3 independent experiments). *p*‐values were determined by one‐way ANOVA with the Tukey multiple comparison test. ****p < 0.0001. G) qRT–PCR results demonstrating the relative expression levels of the DEGs and TFs for SSECs and GECs identified by scRNA‐seq, including *KRT14*, *S100A8*, *CLDN3* and *TP63*, in CC cell lines (2D‐SiHa/2D‐HeLa), 3D organoids (3D‐SiHa/3D‐HeLa) and tissue samples (10 SCC samples and 10 ADC samples). Error bar: mean value ± SD (*n* = 3 independent experiments). *p*‐values were determined by two‐sided Student *t*‐test or Mann–Whitney test. *p < 0.05; **p < 0.01; ***p < 0.001; ****p < 0.0001. CC, cervical cancer; scRNA‐seq, single‐cell RNA sequencing; CAFs, cancer‐associated fibroblasts; ADC, cervical adenocarcinoma; SCC, squamous cell carcinoma; DAPI, 4′,6‐diamidino‐2‐phenylindole; qRT‐PCR, real‐time quantitative reverse transcription polymerase chain reaction; SD, standard deviation; ANOVA, analysis of variance; DEGs, differentially expressed genes; SSECs, stratified squamous epithelial cells; GECs, glandular epithelial cells.

To validate the features and functions of structural cells identified by the aforementioned scRNA‐seq analysis, we used a 3D organoid culture (**Figure** **3**A). Given that 3D CC organoids could not be cultivated by the traditional CC cell line alone, such as SiHa or HeLa (Figure 3B), we assumed that the nutritional and structural support from the stroma, including endothelial cells and fibroblasts, would assist in the establishment of 3D CC organoids. Based on the cell proportions observed in the CC samples through immunohistochemistry (IHC) staining (Figure S2D, Supporting Information), we mixed SiHa‐GFP/ HeLa‐GFP with HUVECs‐mCherry and CAFs at a ratio of 9:1:3 and successfully generated biomimetic CC organoids modelling solid tumors (Figure 3C,D), which further proved the indispensable roles of structural cells in TME. Intriguingly, CC spheroid organoids could invade Matrigel (Figure 3E), upregulating invasion‐related genes^[^ ^27^, ^28^ ^]^ (*CDC42*, *RHOA*, *ROCK1*, and *MMP7)*. Moreover, expression of these genes was more remarkable in 3D invasive CC organoids than in immortalized tumor cell lines cultured in traditional 2D conditions without endothelial cells or CAFs (Figure 3F), confirming the protumoral effects of cancer stroma^[^ ^29^ ^]^ and the invasive property of the CC organoids.

Furthermore, we verified the expression of the DEGs and TFs of SSECs and GECs identified by scRNA‐seq, including *KRT14*, *S100A8*, *CLDN3* and *TP63* in CC cell lines, organoids and tissue samples. Of note, the differences in gene expression between SCC and ADC were generally more obvious in the 3D organoids than in the traditional 2D cell lines, and these differences were more similar to those observed in tissue samples, highlighting both the biomimetic potential of the 3D organoids and the reliability and credibility of our scRNA‐seq results (Figure 3G). In summary, using 3D organoids, we confirmed the promalignant properties of structural cells and validated the scRNA‐seq results of DEGs and TFs in SCC and ADC.

### 2.3 Immune Cells: Regulating the Complicated Tumor Ecosystem

#### 2.3.1 Myeloid Cells: more than Antigen Processing and Presentation

Myeloid cells, including monocytes, macrophages and dendritic cells (DCs), which serve as bridges between the innate and adaptive immune system, were subsequently investigated.

**Figure 4:**
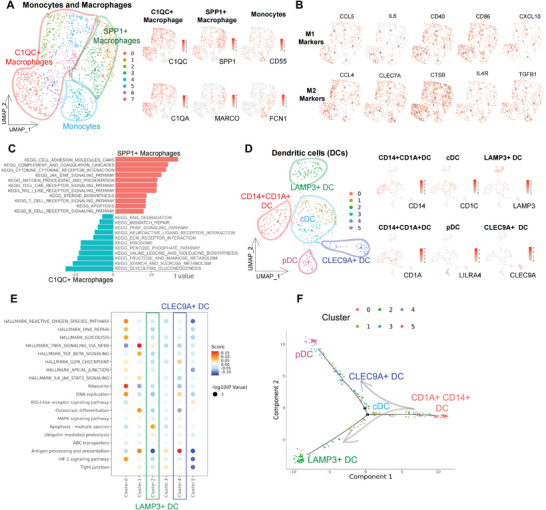
Myeloid cells differentiate into monocytes, macrophages, and dendritic cells with distinguished roles. A) UMAP projection of the eight subclusters of myeloid cells generated from unsupervised clustering, which could be categorized into three main groups (left) based on marker gene expression (right): *CD55* and *FCN1* for monocytes (subcluster 4); *C1QC* and *C1QA* for C1QC+ macrophages (subclusters 0, 2, 5, 6, and 7); *SPP1* and *MARCO* for SPP1+ macrophages (subclusters 1 and 3). Color key from gray to red indicates relative expression levels from low to high. B) Traditional marker expression for M1‐type macrophages (*CCL5*, *IL6*, *CD40*, *CD86*, and *CXCL10*) and M2‐type macrophages (*CCL4*, *CLEC7A*, *CTSB*, *IL4R*, and *TGFB1*) plotted onto UMAP projection. C) Bar plot of the enriched KEGG pathways of C1QC+ and SPP1+ macrophages presented with a *t*‐value of the GSVA score (SPP1+ macrophages versus C1QC+ macrophages). D) UMAP projection of the six subclusters of DCs generated from unsupervised clustering, which could be categorized into five main groups (left) based on marker gene expression (right): *CD14*, *CD1A* for CD14+CD1A+ DCs (subcluster 0); *CD1C* for cDCs (subclusters 1 and 3); *LMAP3* for LAMP3+ DCs (subcluster 2); *CLEC9A* for CLEC9A+ DCs (subcluster 4); and *LILRA4* for pDCs (subcluster 5). E) Bubble plot of the enriched pathways of each DC cluster through QUSAGE analysis presented with statistical significance [−Log10(*p*‐value)] and the enrichment score (color key from blue to red). F) Differentiation trajectory of DC subclusters predicted by monocle. KEGG, Kyoto Encyclopedia of Genes and Genomes; GSVA, gene set variation analysis; DCs, dendritic cells.

Monocytes and macrophages were reclustered and classified into the following three major subgroups^[^ ^30^ ^]^ (**Figure** **4**A): 1) monocytes (*CD55* and *FCN1*); 2) C1QC+ macrophages (*C1QC* and *C1QA*;); and 3) SPP1+ macrophages (*SPP1* and *MARCO*). Although macrophages have traditionally been classified as proinflammatory (M1‐like) or anti‐inflammatory (M2‐like) macrophages, this classic dichotomy was not applicable in our study, failing to discriminate the cells into distinct states (Figure 4B). Gene set variation analysis (GSVA) (Figure 4C) indicated that the C1QC+ macrophages were extensively engaged in the immune response (Toll‐like receptor signaling pathway, antigen processing and presentation). In contrast, SPP1+ macrophages were closely associated with ECM‐receptor interactions, substance transport, and metabolism. Collectively, the SPP1/C1QC dichotomy potentially outperformed the classic M1/M2 classification, better illustrating the heterogeneity in the molecular features and function of macrophages in CC TME.

DCs, as professional antigen‐presenting cells, were reclustered and categorized into five groups^[^ ^31^ ^]^ (Figure 4D): 1) CD14+CD1A+ DCs (*CD14* and *CD1A*); 2) cDCs (*CD1C*); 3) LAMP3+ DCs (*LAMP3*); 4) CLEC9A+ DCs (*CLEC9A*); and 5) pDCs (*LILRA4*). Functional analysis indicated that CLEC9A+ DCs and cDCs participated in antigen processing and presentation, whereas LAMP3+ DCs were associated with apoptosis (Figure 4E). Next, we inferred the developmental trajectories of DCs (Figure 4F): CD14+CD1A+ DCs transformed into pDCs and cDCs, which gradually diverged into two paths: CLEC9A+DCs and LAMP3+DCs. Therefore, to activate antigen presentation of DCs to the greatest extent would be a breakthrough point for CC immunotherapy.

#### 2.3.2 CD4+ and CD8+ T Cells: Revealing Immunosuppression and Exhaustion in CC

**Figure 5:**
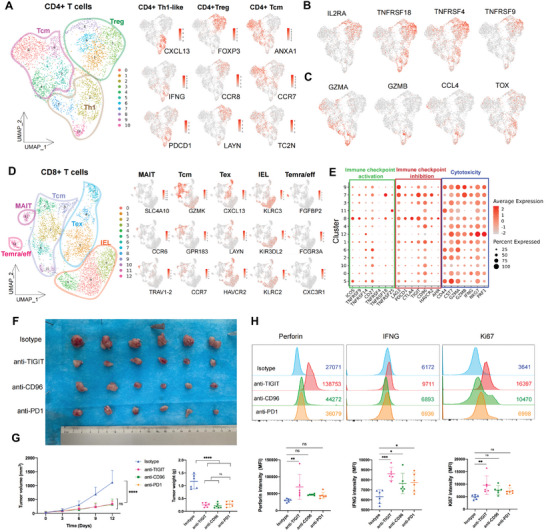
CD8+ and CD4+ T cells involved in immunosuppression and exhaustion in CC. A) UMAP projection of the 11 clusters of CD4+ T cells generated from unsupervised reclustering (left), which could be further categorized into three major groups according to marker gene expression (right): *CXCL13*, *IFNG*, *PDCD1* for Th1‐like cells (subcluster 2, 7, 10); *FOXP3*, *CCR8*, *LAYN* for Tregs (subcluster 1, 3, 6, 8); *CCR7*, *ANXA1*, *TC2N* for Tcm cells (subcluster 0, 4, 5, 9). Color key from gray to red indicates relative expression levels from low to high. B) Expression levels of *IL2RA*, *TNFRSF18*, *TNFRSF4*, and *TNFRSF9* in CD4+ T cells are plotted onto the UMAP projection. Color key from gray to red indicates relative expression levels from low to high. C) Expression levels of *GZMA*, *GZMB*, *CCL4*, and *TOX* in CD4+ T cells are plotted onto the UMAP projection. Color key from gray to red indicates relative expression levels from low to high. D) UMAP projection of the 13 subclusters of CD8+ T cells generated from unsupervised reclustering, which could be categorized into five main groups (left) based on marker gene expression (right): *SLC4A10*, *CCR6*, *TRAV1‐2* for MAITs (subcluster 11); *GPR183*, *CCR7*, *GZ*MK for Tcms (subclusters 2, 5, 6, 9, and 10); *LAYN*, *CXCL13*, *HAVCR2* for Tex cells (subclusters 1, 7, and 8); *KLRC2*, *KLRC3*, *KIR3DL2* for IELs (subclusters 0, 3, 4, and 10); *FGFBP2*, *FCGR3A*, *CXC3R1* for Temra/effffs (subcluster 12). Color key from gray to red indicates relative expression levels from low to high. E) Dot plot indicating the average expression levels and cell expressing proportion of markers for immune checkpoint activation, immune checkpoint inhibition, and cytotoxicity among different CD8+ T cell clusters. The colors represent the average expression levels, and dot sizes represent the percentage expression of selected genes. F) Photographs of TC1 tumors by indicated treatment. The tumors were removed from C57BL/6 mice at day 12 after TC1 cell injection (*n* = 6 for each group: isotypes, anti‐TIGIT, anti‐CD96, anti‐PD1). G) Average tumor growth curves of TC1 tumors in mice with different treatment options (left) and the scatter plot indicating the tumor weights in different treatment groups (right). Error bar: mean value ± SD (*n* = 6 for each treatment group). *p*‐values were obtained by one‐way ANOVA with the Tukey multiple comparison test. **p < 0.01, ****p < 0.0001, ns not significant. H) (Upper) Representative flow cytometric histograms and (lower) the scatter plots of the MFI showing perforin, IFNG and Ki67 expression in T cells in the TC1 tumors after different treatment options. Error bar: mean value ± SD (*n* = 6 for each treatment group). *p*‐values were obtained by one‐way ANOVA with the Tukey multiple comparison test. *p < 0.05; **p < 0.01; ***p < 0.001; ns, not significant. CC, cervical cancer; IELs, intraepithelial lymphocytes; SD, standard deviation; ANOVA, analysis of variance; MFI, median fluorescence intensity.

To determine the exact roles of CD4+ T cells in the CC TME, we performed unsupervised clustering and categorized 11 subclusters into three main groups^[^ ^32^ ^]^ (**Figure** **5**A): 1) Tcms (*CCR7*, *ANXA1*, *TC2N*); 2) type I helper‐like T cells (Th1‐like cells) (*CXCL13*, *IFNG*, *PDCD1*); and 3) regulatory T cells (Tregs) (*FOXP3*, *CCR8*, *LAYN*). Notably, high expression of the chemokine receptor *CCR8*, which mediates recruitment, along with a regulatory marker (*IL2RA*) and co‐stimulatory molecules (*TNFRSF18*, *TNFRSF4*, and *TNFRSF9*) were observed in CD4+ Tregs (Figure 5B). In contrast, Th1‐like CD4+ T cells, characterized by the expression of *CCL4*, *GZMA* and *GZMB* were found to be involved in viral protein interactions, natural killer cell‐mediated cytotoxicity and the T cell receptor signaling pathway (Figure S3A, Supporting Information), revealing their positive roles in tumor immune defense. Moreover, we unexpectedly noticed that *TOX*, a well‐known marker for exhaustion, was also expressed in Th1‐like cells, indicating a dysfunctional state (Figure 5C). To summarize, CD4+ Tregs recruited to the CC TME exhibited inhibitory immune functions, whereas CD4+ Th1 cells demonstrated a positive immune response but showed signs of dysregulation and exhaustion.

CD8+ T cells are not only key components in the TIME but also essential elements of cancer immunotherapy. Herein, we performed unsupervised clustering of CD8+ T cells yielding 13 subclusters and classified them into the following five main groups^[^ ^33^ ^]^ (Figure 5D): 1) mucosa‐associated invariant T cells (MAITs) (*SLC4A10*, *CCR6*, *TRAV1‐2*); 2) central memory T (Tcm) cells (*GPR183*, *CCR7*, *GZMK*); 3) exhausted T (Tex) cells (*LAYN*, *CXCL13*, *HAVCR2*); 4) intraepithelial lymphocytes (IELs) (*KLRC2*, *KLRC3*, *KIR3DL2*); and 5) recently activated effector memory or effector T (Temra/eff) cells (*FGFBP2*, *FCGR3A*, *CXC3R1*). Next, we evaluated the functional state of CD8+ T cells (Figure 5E). *NKG7* and *PRF1* were specifically highly expressed in Temra/eff cells (subcluster 12), which exhibited a positive functional state. Additionally, the proportion of cells with activation checkpoint expression was generally low, whereas inhibition checkpoint expression was clearly observed in CD8+ Tex cells (subclusters 1, 7, and 8), which lay at the end of the differentiation trajectory of CD8+ T cells according to pseudo‐time analysis (Figure S3B, Supporting Information), highlighting the immune dysregulation and dysfunction of CD8+ Tex cells. Notably, the higher and broader expression of *TIGIT* and *CD96* than *PDCD1* and *CTLA4* indicated that these immune checkpoints might also mediate CD8+ T cell exhaustion, holding great potential for developing novel ICB therapies for clinical use. Furthermore, we established tumor‐bearing mouse models and proved that anti‐TIGIT, anti‐CD96, and anti‐PD1 could remarkably inhibit the growth of CC cells in vivo; yet no statistical differences were found between these three groups (Figure 5F,G). Moreover, we observed proliferation of T cells featuring Ki67 expression and enhanced anti‐tumor immunity featuring perforin and IFNG upregulation within the TIME after ICB treatment, especially in the anti‐TIGIT group (Figure 5H). Based on the aforementioned findings, we supposed that anti‐TIGIT and anti‐CD96 could restore the anti‐tumor immunity of CD8+ T cells, without inferiority to anti‐PD1, presenting unprecedented potential for them to become ICB targets for clinical use.

#### 2.3.3 TCR Clonotype Expansion in CC: Three Immune Reaction Patterns Mediated by T Cells

**Figure 6:**
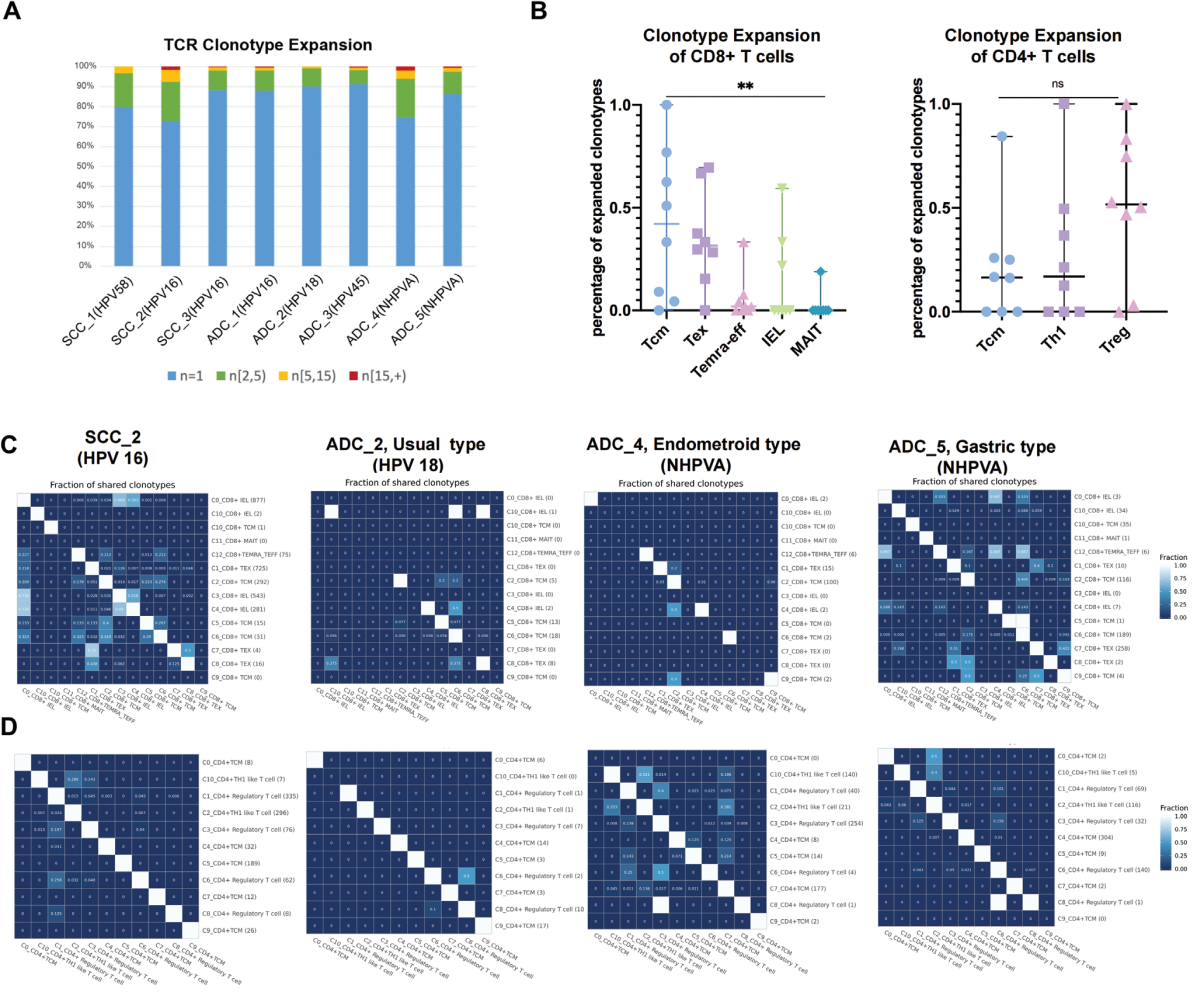
TCR clonotype expansion in CC: three immune reaction patterns. A) Bar plot describing the TCR clonotype expansion in each CC sample in four categories: *n* = 1, 2 ≤ *n* <5, 5 ≤ *n* <15, and *n* > 15. B) Scatter plots indicating the proportions of five subclusters of CD8+ T cells (left) and 3 subclusters of CD4+ T cells (right) with expanded TCR clonotypes (*n* ≥ 2). Error bar: median value with 95% CI (*n* = 8 standing for 8 CC samples). *p*‐values were obtained by the Kruskal–Wallis test. **p < 0.01; ns, not significant. C) Heat maps of clonotype sharing within different CD8+ T cell subclusters in representative samples including HPV‐positive SCC (SCC_2, HPV 16 positive), HPVA ADC (ADC_2, HPV 18 positive), and NHPVA ADC (ADC_4, endometrioid type and ADC_5, gastric type). Color key from blue to white indicates the shared fraction from low to high. D) Heat maps of clonotype sharing within different CD4+ T cell subclusters in representative samples including HPV‐positive SCC (SCC_2, HPV 16 positive), HPVA ADC (ADC_2, HPV 18 positive), and NHPVA ADC (ADC_4, endometrioid type and ADC_5, gastric type). Color key from blue to white indicates the shared fraction from low to high. TCR, T‐cell receptor; CC, cervical cancer; CI, confidence interval; HPV, human papillomavirus; ADC, cervical adenocarcinoma; SCC, squamous cell carcinoma; NHPVA, non‐human papillomavirus‐associated.

Using scRNA‐seq and TCR‐seq, we comprehensively illustrated the clonal expansion of T cells (**Figure** **6**A). We found that HPV‐positive SCC samples generally harbored a higher degree of expanded clonotypes than HPVA ADC samples. Nevertheless, expanded clonotypes were also enriched in NHPVA ADC samples, probably because of the activation of cancer neoantigens, even without HPV infection.

We also investigated clonotype expansion in various CD8+ T cells and CD4+ T cell subclusters (Figure 6B). Notably, CD8+ Tcm and CD8+ Tex cells were the clusters with the highest extent of expanded clonotypes, followed by CD8+ IELs, whereas TCR clonotypes were dramatically less expanded in effector T (Temra/eff) cells. These findings imply that a large proportion of CD8+ T cells in the CC TME are either maintained in quiescence as CD8+ Tcm cells or exhibit functional dysregulation as CD8+ Tex cells. Regarding CD4+ T cells, Tregs harbored the highest extent of expanded clonotypes, with a median proportion of 50%, revealing great proliferation and expansion, highlighting their inhibitory effect on the TME of CC. Collectively, the clonotype expansion of CD8+ T cells and CD4+ T cells suggests an immunosuppressive state in the TME.

Subsequently, we identified three immune reaction patterns on the basis of TCR clonotypes overlapping within CD8+ and CD4+ T cell clusters in CC samples with different HPV infection statuses, namely coordinated immune reaction (CIR), inactive immune reaction (IIR) and imbalanced immune reaction (IBIR) between CD4+ and CD8+ T cells (Figure 6C,D). Specifically: 1) in those samples infected with HPV16 (represented by SCC_2), the TCR clonotypes greatly overlapped across various CD8 +T cell subsets (Tcm, IEL, Temra/eff, and Tex cells) and CD4+ T cell subsets (Tcm, Treg, and Th1‐like cells), highlighting a coordinated immune response; 2) for samples infected with other subtypes of HPV such as HPV18 (represented by ADC_2), we observed much fewer overlapping TCR clonotypes within both CD8+ T cell clusters and CD4+ T cell clusters, demonstrating an inactive state of immune function; and 3) in NHPVA ADC, we found an imbalanced immune response between CD8+ T and CD4+ T cells, which might explain the severe malignancy of this histologic subtype. More specifically, the gastric‐type sample (ADC_5) demonstrated extensive overlapping TCR clonotypes within CD8+ Tex, Temra/eff, Tcm cells, and IELs but less interaction between distinct CD4+ T cell subsets. Yet in the endometrioid‐type ADC (ADC_4), we noticed highly overlapped TCR clonotypes within CD4+ Tcm, Tregs and Th1‐like cells, but rarely shared clonotypes in CD8+ T cells. The aforementioned findings not only explain why ADC, especially NHPVA ADC, generally presents a more malignant phenotype than SCC from the perspective of T lymphocyte‐mediated immune reaction upon different HPV infection statuses but also indicate great potential for applying this immune response pattern to predict immune therapy efficacy and disease prognosis.

### 2.4 Unique Cellular Interactions in HPV‐Positive SCC, HPVA ADC, and NHPVA ADC

**Figure 7:**
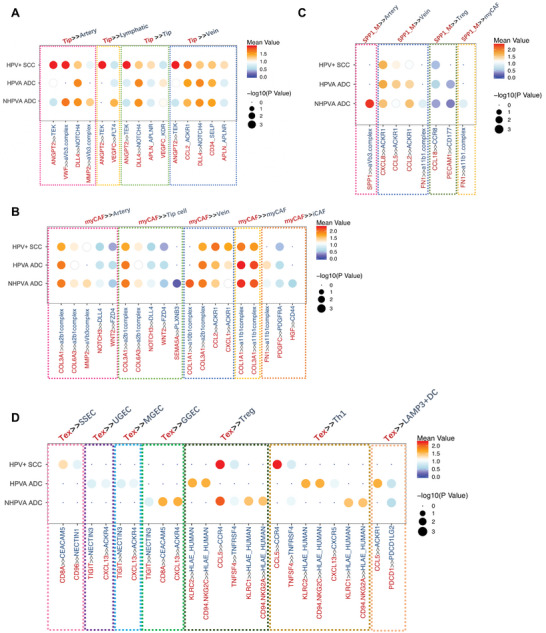
Ligand–receptor interactions in CC with different histologic subtypes and HPV infection statuses. A) Dot plots demonstrate selected ligand–receptor interactions between tip cells and other endothelial cells, respectively, in HPV‐positive SCC, HPVA ADC and NHPVA ADC. The ligand–receptor interactions and histologic subtypes are indicated by the columns and rows, respectively. The means of the average expression levels of two interacting molecules are indicated by color key, with blue to red representing low to high expression. The [−Log10(*p*‐values)] were indicated by dot size. Tip cells contacted each other through DLL4‐NOTCH interaction and attracted other lymphovascular endothelial cells by producing angiogenic factors such as APLN, ANGPT2, and VEGFC. Intriguingly, ANGPT2>>TEK and APLN>>APLNR interactions were more prominent in the HPV‐positive SCC microenvironment, while VEGFC>>FLT4 and DLL4>>NOTCH4 crosstalk was more remarkable in the ADC ecosystem, especially in NHPVA ADC. Additionally, tip cells characteristically specifically expressing MMP2 in NHPVA ADC, reflect a greater extent of angiogenesis, which was in accordance with a more malignant phenotype. B) Dot plots demonstrate selected ligand–receptor interactions between myCAFs and other cell types respectively in HPV‐positive SCC, HPVA ADC, and NHPVA ADC. MyCAFs show extensive engagement in ECM receptor interactions (collagen>>integrin, WNT>>FZD), illustrating their important roles in ECM remodeling. Moreover, SEMA5A–PLXNB3 interaction between myCAFs and tip cells merely exist in NHPVA ADC samples, which is involved in axon guidance, partly explaining the high rate of neural invasion in this histologic subtype. In addition, we found that growth factors also took part in the cellular crosstalk between myCAFs and iCAFs such as PDGFC and HGF; yet HGF>>CD44 specifically existed in NHPVA ADC microenvironment. C) Dot plots demonstrate selected ligand–receptor interactions between SPP1+ macrophages and other cell types, respectively, in HPV‐positive SCC, HPVA ADC and NHPVA ADC. SPP1+ macrophages produced and responded to certain chemokines (CXCL8/CCL5/CCL2>>ACKR1, CCL18>>CCR8, etc.), among which the widely existed CCL18>>CCR8 interaction might mediate the attraction of CD4+ Tregs. Moreover, SPP1+ macrophages interacted with stroma cells mainly through cell adhesion. And we noticed that SPP1>>avb3 complex and FN1>>a11b1 complex were more remarkable in the NHPVA ADC microenvironment, indicating a greater infiltration of SPP1+ macrophages. D) Dot plots demonstrate selected ligand–receptor interactions between CD8+ Tex cells and other cell types respectively in HPV‐positive SCC, HPVA ADC, and NHPVA ADC. CD8+ T ex cells show comprehensive crosstalk with malignant epithelial cells, DC cells and CD4+ T cells. Notably, the cellular crosstalk between CD8+ Tex cells and epithelial cell were associated with chemotaxis (CXCL13>>ACKR4), antigen‐recognition (CD8A>>CEACAM5) and immune dysfunction (CD96>>NECTIN1, TIGIT>>NECTIN3). Remarkably, CD96‐NECTIN1 interaction was found within CD8+ T ex cells and SSECs, whereas TIGIT‐NECTIN3 interaction was demonstrated within CD8+ T ex cells and GECs, which suggested that different immune checkpoint blockades could be considered for SCC and ADC. Besides, we noticed a greater HLA‐E mediated immune inhibitory response between CD8+ T cells and CD4+ T cells in ADC microenvironment, which might contribute to progressive loss of effector functions of tumor‐specific T cells, a state known as cell exhaustion. Furthermore, regarding the cellular crosstalk between CD8+ T ex cells and DCs, PDCD1–PDCD1LG2 interaction, inducing programmed death of T cells, was observed merely in ADC samples, not in SCC samples, which manifested a more severe immune‐repressed status of ADC. CC, cervical cancer; HPV, human papillomavirus; SCC, squamous cell carcinoma; HPVA, human papillomavirus‐associated; ADC, cervical adenocarcinoma; NHPVA, non‐human papillomavirus‐associated; myCAFs, myoblastic cancer‐associated fibroblasts; ECM, iCAFs, inflammatory cancer‐associated fibroblasts; DC, dendritic cell; SSECs, stratified squamous epithelial cells.

Having dissected the diverse cell components in the TME of CC, we further investigated the cell–cell interaction network and illustrated the CC TME using schematic diagrams (Figure S4, Supporting Information). Given that sprouting angiogenesis, ECM remodeling and immune escape are significant hallmarks of cancer, we elucidated the marked cell–cell interactions of the following four representative cell types in HPV‐positive SCC, HPVA ADC and NHPVA ADC: tip cells (**Figure** **7**A), myCAFs (Figure 7B), SPP1+ macrophages (Figure 7C), and CD8+ Tex cells (Figure 7D). In brief, we discovered that ADC and SCC differed in the TIME. For instance, the PDCD1‐PDCD1LG2 interaction between CD8+ Tex cells and LAMP3+ DCs, which mediates tumor immune escape, was prominent in ADC samples. In addition, CD96‐NECTIN1 and TIGIT‐NECTIN3 interactions were observed between CD8+ Tex cells, SSECs and GECs, potentially indicating different patterns of immune checkpoint expression in SCC and ADC. In NHPVA ADC, we discovered remarkable involvement of MMP2 in tumor angiogenesis, and the SEMA5A–PLXNB3 interaction was possibly related to neural invasion. These novel findings explain the aggressiveness of NHPVA ADC compared with the other two histologic subtypes.

### 2.5 Novel Diagnostic and Prognostic Biomarkers as well as Potential Therapy Targets Were Established by Integration of scRNA‐seq, the TCGA Dataset, and Clinical CC Samples

**Figure 8:**
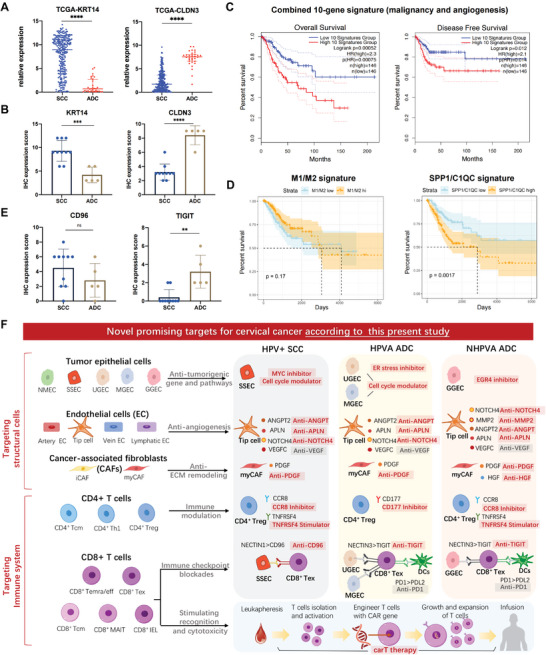
Novel diagnostic and prognostic biomarkers for CC and potential therapy targets based on different histologic subtypes and HPV infection statuses. A) Scatter plots indicating the expression of *KRT14* and *CLDN3* between 257 SCC samples and 32 ADC samples in the TCGA CESC dataset. Error bar: median value with interquartile range. *p*‐values were obtained by the two‐sided Mann–Whitney test. ****p < 0.0001. B) IHC expression scores of *KRT14* and *CLDN3* in 10 SCC samples and 5 ADC samples. Error bar: mean value with SD. *p*‐values were obtained by the two‐sided unpaired test. ***p < 0.001, ****p < 0.0001. C) Kaplan–Meier curves illustrating the prognostic value of the combined 10‐gene signature indicating both epithelial malignancy (*KRT17*, *S100A10*, *CDKN2A*, *CLIC1*, *KRT20*) and sprouting angiogenesis (*ESM1*, *ADAMTSL2*, *COLA1*, *APLN*, *ANGPTL2*), constructed by the web tool Gepia2 with the CESC TCGA dataset: the higher the signature expression was, the shorter the OS and DFS were (*p* = 0.00052 and 0.012, respectively by the log rank test). D) Kaplan–Meier curves indicating the prognostic value of the SPP1/C1QC signature embodying macrophages immunoregulation superior to the M1/M2 signature (*p* = 0.17): the higher SPP1/C1QC signature expression was, the shorter OS would be (*p* = 0.0017 by log rank test). E) IHC expression scores of CD96 and TIGIT in 10 SCC samples and 5 ADC samples. Error bar: mean value with SD. *p*‐values were obtained by two‐sided unpaired test. **p < 0.01; ns, not significant. F) Novel potential targets for precise treatment uniquely to HPV‐positive SCC, HPVA ADC, and NHPVA ADC. CC, cervical cancer; HPV, human papillomavirus; TCGA, The Cancer Genome Atlas; CESC, cervical and endocervical cancer; IHC, immunohistochemistry; SD, standard deviation; OS, overall survival; DFS, disease‐free survival; SCC, squamous cell carcinoma; HPVA, human papillomavirus‐associated; NHPVA, non‐human papillomavirus‐associated.

Given that ADCs would be misdiagnosed because of occult lesions, inappropriate sampling and ambiguous judgement,^[^ ^34^ ^]^ we wondered whether the DEGs between squamous epithelial cells and glandular cells, previously identified from scRNA‐seq and would be the biomarkers to distinguish ADC from SCC. As the expression of *KRT14* and *CLDN3* showed high consistency in both scRNA‐seq and 3D organoids, exhibiting clinical value, we further verified their heterogenous expression using TCGA cervical and endocervical cancer (CESC) dataset (**Figure** **8**A) and clinical samples from our center (Figure 3G). We examined the *KRT14* and *CLDN3* expression by immunohistochemistry (IHC) staining in SCC and ADC tissues. As expected, *KRT14* had a wider expression in SCCs while *CLDN3* had a stronger expression in ADCs (Figure 8B and Figure S5A,B, Supporting Information), indicating their great potential to become diagnostic biomarkers respectively for SCC and ADC.

Next, since unique cell clusters such as malignant epithelial cells and tip cells play crucial roles in CC TME, we wondered whether the marker gene expression of these cell clusters could offer prognostic value. Therefore, we established novel gene signatures using the TCGA CESC dataset. Notably, the malignant epithelial gene signature (Figure S5C, Supporting Information), the tip cell gene signature (Figure S5D, Supporting Information) and a combined gene signature (Figure 8C) embodying cancer hallmarks, including sustaining proliferation and vasculature inducement, exhibited promising prognostic value for predicting overall survival (OS) and disease‐free survival (DFS). Additionally, to determine whether the novel SPP1/C1QC dichotomy of macrophages was clinically informative, we established both the SPP1/C1QC and M1/M2 signatures (Figure 8D). Remarkably, a higher SPP1/C1QC signature demonstrated poorer survival (*p* = 0.0017), superior to the M1/M2 signature which was unhelpful for evaluating prognosis (*p* = 0.17).

Finally, we proposed promising novel treatment targets for CC that are unique to HPV‐positive SCC, HPVA‐ADC, and NHPVA‐ADC (Figure 8F): 1) regarding epithelial cell‐targeted therapies, we considered cyclin‐dependent kinase (CDK) inhibitors for HPV‐positive CC, since HPV infection contributes to cell cycle overactivation, thus leading to tumorigenesis. We also suggested MYC inhibitors for HPV‐positive SCC, ER stress inhibitors for usual‐type ADC and ERG4 inhibitors for gastric‐type ADC, given the dominant roles of *MYC*, *DDIT3* and *ERG4* in regulating SSECs, UGECs and GGECs, respectively; 2) regarding tip cell‐targeted therapies, we proposed blocking *VEGFC*, *ANGPT2*, *APLN* and *NOTCH4*, which are commonly expressed in tip cells in CC. Of note, MMP2 inhibitors should be further investigated in NHPVA ADC, since *MMP2* was found to be highly expressed by tip cells in such aggressive histologic subtypes; 3) regarding fibroblast‐targeted therapies, we proposed using anti‐PDGF in both SCC and ADC, and anti‐HGF specifically in NHPVA ADC; 4) regarding CD4+ Treg‐targeted treatment, we proposed anti‐CCR8/CD177 treatment to inhibit the infiltration of CD4+ Tregs, given that *CCR8* and *CD177* mediate the migration and activation of CD4+ Tregs. Moreover, we proposed *TNFRSF4* (a co‐stimulatory molecule) stimulation in both HPV‐positive SCC and NHPVA ADC because *TNFRSF4* stimulation may inhibit the suppressive effect of CD4+ Tregs;^[^ ^35^ ^]^ and 5) regarding CD8+ T cell‐targeted therapies, we assumed that PD1/PDL1 agents should be more actively used in ADC. Besides, targeting *CD96* for SCC and *TIGIT* for ADC should also be further investigated, since we have preliminarily proved that these two immune checkpoints were differentially expressed in different histologic subtypes of CC (Figure 8E and Figure S4E,F, Supporting Information). Further research on TCR epitopes targeting HPV and neoantigens of NHPVA CC would be extremely valuable for the development of chimeric antigen receptor (CAR) T cell immunotherapy. Collectively, these treatment strategies offer prospective options for the currently limited targeted therapies available in clinical practice (vascular endothelial growth factor [VEGF] blockade and anti‐PD/PDCD1 agents), hopefully paving the way for precise and personalized treatment of CC in the future.

## 3 Discussion

**Figure 9:**
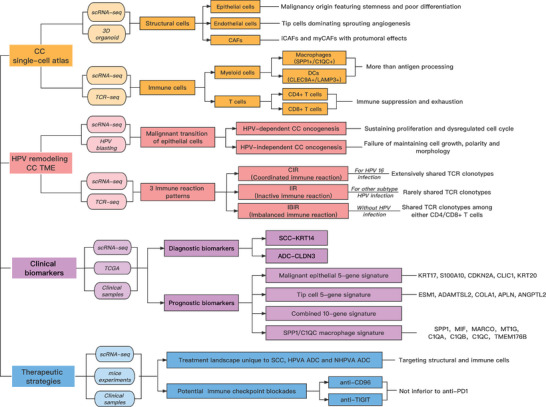
Flow chart of the current study demonstrating the main methods and significant findings.

In the field of CC, previous studies mainly focused on the heterogeneity of cervical cancer in different clinical stages, with limited sample size. Here, for the first time, we generated a single‐cell transcriptional atlas of eight CC samples with different histologic subtypes and HPV infection statuses. Through the combination of scRNA‐seq and 3D organoid culture, we demonstrated the significance of structural cells in the CC TME, among which epithelial cells mediated oncogenesis, tip cells dominated sprouting angiogenesis and CAFs assisted tumor progression. From the perspective of HPV infection, we proposed innovative mechanisms of HPV remodeling CC TME. Notably, we identified three immune reaction patterns in CC patients with different HPV infection statuses by integrating scRNA‐seq and TCR‐seq. Based on bioinformatics analysis, we established novel diagnostic and prognostic biomarkers for CC by verifying the TCGA CESC dataset and clinical samples from our center. Lastly, we pioneer by proposing a precise treatment landscape uniquely for HPV‐positive SCC, HPVA ADC and NHPVA ADC, which will be of tremendous clinical value (**Figure** **9**).

Epithelial cells, endothelial cells and fibroblasts are essential structural components of the TME that serve as barriers, form the lining of vessels and make up the stroma. In this study, we thoroughly investigated these three cell types: 1) in terms of epithelial cells, we illustrated the potential malignant origin of epithelial cells featuring stemness and poor differentiation, as well as the potential mechanism of HPVA and NHPVA CC oncogenesis. Notably, we found that malignant epithelial cells were derived from different individuals, whereas non‐malignant epithelial cells featuring *SOX17* expression were shared, which was similar to the finding of a previous research on tumor heterogeneity.^[^ ^36^ ^]^ Our findings also highlight the importance of a deeper investigation of NMECs in suppressing CC oncogenesis possibly mediated by *SOX17* through the WNT/*β*‐catenin pathway,^[^ ^37^, ^38^ ^]^ which might be valuable for treatment development; 2) since angiogenesis is one of the hallmarks of cancer, we studied the lymphovascular system in CC and discovered tip cells. Remarkably, a high abundance of tip cells indicated shorter survival, in accordance with previous research on lung tumor tip cells.^[^ ^25^ ^]^ More significantly, this previous study showed that tip cells were the most sensitive to anti‐VEGF blockade among the various endothelial cell clusters. Therefore, we suggest that tip cells could serve not only as prognostic biomarkers but also as promising anti‐angiogenic targets for CC. In addition to anti‐VEGF treatment, other anti‐angiogenic targets should also be studied, such as anti‐ANGPT, anti‐APLN, andti‐NOTCH4 and anti‐MMP2; and 3) inspired by the research of Chen et al.^[^ ^39^ ^]^ indicating the crucial function of CAFs, we identified two types of CAFs: iCAFs mediating inflammation‐to‐cancer transformation through the interaction of cytokines and chemokines, and myCAFs engaged in ECM remodeling. These two types of CAFs produce different patterns of growth factors to stimulate tumor proliferation. Moreover, myCAFs in subcluster 9 were involved in antigen processing and presentation pathways, which is partly consistent with the findings of Elyada et al.’s study,^[^ ^40^ ^]^ which first reported antigen‐presenting CAFs. These findings indicate that CAFs play multiple roles in TME and may serve as therapeutic targets to slow CC progression. Collectively, we not only illustrated the critical roles of structural cells in the CC TME with differences between SCC and ADC but also identified key genes and pathways in structural cells, generating novel prognostic biomarkers reflecting cancer hallmarks and promising therapeutic targets for CC.

Considering the necessity of structural cells in the TME and that 3D organoids can exhibit more heterogeneity than traditional cell lines, we generated a CC organoid consisting of tumor epithelial cells, endothelial cells and fibroblasts, and preliminarily verified that such an organoid could manifest the key molecular and functional properties of structural cells discovered from scRNA‐seq. Therefore, we propose a novel research model that integrates high‐throughput sequencing with organoid culture technology to build more biomimetic in vitro models and validate the in‐silico analysis results.

In addition to structural cells, immune cells were also a focus of our research. Regarding macrophages, we discovered that the SPP1/C1QC dichotomy of macrophages was more reasonable than the classic M1/M2 classification in CC, further supporting previous studies.^[^ ^30^, ^41^ ^]^ In terms of DCs, we identified that CLEC9A+ DCs exerted positive antigen presentation, while LAMP3C+ DCs were related to apoptosis, which implied a mature and exhaustion‐like functional state concordant with previous researches.^[^ ^42^, ^43^ ^]^ Notably, we discovered CD8+ T ex cells expressing immune checkpoints, including not only *PDCD1* but also *TIGIT* and *CD96*, which have already aroused great interests as novel immune checkpoint targets.^[^ ^44^, ^45^ ^]^ Furthermore, we conducted TCR‐seq, which has been widely used in various immune‐related diseases, including cancer, but is seldom used for CC. For the first time, we found that HPV16 stimulated much more clonal expansion than other high‐risk HPV subtypes, such as HPV18 and HPV45, while the neoantigens in NHPVA ADC might also contribute to T cell proliferation and interaction; yet, there was an imbalanced immune response between CD8+ and CD4+ T cells. These findings provide new insights into the development of CC immunotherapy, including anti‐tumor vaccines and chimeric antigen receptor (CAR) T cell therapy.

Regarding diagnostic biomarkers, we identified DEGs between SCC and ADC through scRNA‐seq and preliminarily validated that *KRT14* and *CLDN3* could assist in the accurate identification of SCC and ADC. Regarding prognostic biomarkers, we generated several gene signatures that embodied cancer hallmarks in various dimensions, including the malignant epithelial cell gene signature, tip cell gene signature, combined gene signature and SPP1/C1QC macrophage gene signature. To achieve precise treatment for CC, we suggest a unique therapy for HPV‐positive SCC, HPVA ADC and NHPVA ADC, targeting both structural cells and immune cells. For instance, considering the aggressiveness of NHPVA ADC, EGR4, MMP2, and HGF inhibitors should be further investigated. More importantly, regarding immune checkpoints, novel ICB treatments, such as anti‐CD96 and anti‐TIGIT, hold great potential for clinical use, which was proven not to be inferior to anti‐PD1 by in vivo experiments, thus confirming our hypothesis. Additionally, we assumed that anti‐CD96 could be considered in SCC treatment, whereas anti‐TIGIT and anti‐PD1 could be applied in ADC treatment according to the heterogenous expression of the aforementioned immune checkpoints in SCC and ADC tissues.

Although the sample size of our current study was insufficient and further validation of the novel biomarkers and histologic‐specific therapeutic targets in larger sample sizes is required, our findings will facilitate more accurate diagnosis and precise treatment for CC patients with different histologic subtypes and HPV infection statuses.

In conclusion, we depicted, for the first time, a comprehensive CC single‐cell transcriptomic atlas, covering structural cells, immune cells and cellular interactions within both SCC and ADC. Notably, we investigated both HPV‐dependent CC carcinogenesis (attributing to dysregulated cell cycle) and HPV‐independent CC tumorigenesis (attributing to failure to maintain cell growth, polarity, and morphology). Additionally, based on different HPV infection statuses and overlapping TCR clonotype expansion, we proposed three immune reaction patterns, namely, coordinated immune reaction (CIR), inactive immune reaction (IIR) and imbalanced immune reaction (IBIR), consistent with the clinical phenotype of different CC histologic subtypes. More significantly, we discovered novel diagnostics biomarkers (*CLDN3* for ADC and *KRT14* for SCC) with validation of clinical tissues and innovatively established prognostic biomarkers based on several unique cell clusters (malignant epithelial cells, tip cells, and SPP1/C1QC macrophages) embodying cancer hallmarks using TCGA data. Lastly, we not only pioneer by proposing a precise treatment landscape uniquely for HPV‐positive SCC, HPVA ADC and NHPVA ADC but also demonstrated the efficacy and potential of anti‐CD96 and anti‐TIGIT as novel immunotherapy strategies for CC, providing a reliable basis for preclinical studies and hopefully paving the way for the precise treatment of CC.

## 4 Experimental Section

### Human Specimens

The current study was approved by the Ethics Committee of Obstetrics and Gynecology Hospital of Fudan University (2021‐14), and informed consent was obtained from all participants for sample collection. Exclusion criteria were as follows: 1) patients with serious concomitant systemic disorders; 2) history of chemotherapy or radiotherapy before; 3) any history of malignant tumors other than cervical cancer; and 4) patients with evidence of distant metastasis. Three HPV‐positive SCC, three HPVA ADC and two NHPVA ADC samples for scRNA‐seq and TCR‐seq were included, and the HPV infection status of the samples was confirmed by clinical HPV testing and HPV sequence blasting analysis. All eight patients were primarily treated at FIGO stage I‐II preoperatively. Detailed clinical characteristics of the eight CC samples are shown in Table S1, Supporting Information. Another 15 formalin‐fixed and paraffin‐embedded samples (10 SCCs and 5 ADCs; the pathological characteristics are shown in Table S2, Supporting Information) and 20 frozen samples (10 SCC and 10 ADC; the pathological characteristics shown in Table S3, Supporting Information) were collected from the tissue bank of the Obstetrics and Gynecology Hospital of Fudan University (2020‐22).

### Generation of 3D CC Organoids

SiHa, HeLa, and HUVEC cell lines were obtained from American Type Culture Collection. SiHa and HeLa cells were grown in minimum essential medium and RPMI‐1640 supplemented with 10% fetal bovine serum (FBS) (Gibco, Thermo Fisher Scientific), 100 U mL^−1^ of penicillin, 100 mg mL^−1^ of streptomycin, and 250 ng of amphotericin B (NCM Biotech). HUVECs were cultured in an endothelial cell growth medium (Endothelium Cell Medium, ScienCell) supplemented with 5% FBS (Gibco). Primary CAFs were isolated as described previously^[^ ^46^ ^]^ and cultured in the Dulbecco Modified Eagle Medium/F12 (Gibco) supplemented with 10% FBS. All cells were cultured at 37 °C in an atmosphere of 5% carbon dioxide. CS Well 600 chambers (JIYAN, China), which contained 261 wells per chamber, were used to fabricate the 3D organoids. A total of 1000 SiHa/HeLa cells, HUVECs and CAFs per well were mixed in a proportion of approximately 9:3:1, seeded into the chambers and cultured for 48 h to generate organoids.

### Single‐Cell Suspension Preparation

After surgical removal, fresh CC tissue samples were stored in MACS Tissue Storage Solution (Miltenyi Biotec) and subsequently processed according to the manufacturer's instructions in the laboratory of NovelBio Co., Ltd. The samples were first washed with phosphate‐buffered saline (PBS), cut into ≈1 mm^3^ pieces and enzymatically digested using a tumor dissociation kit (Miltenyi Biotec) for 30 min at 37 °C with agitation. After digestion, the dissociated cells were passed through a 70‐µm cell strainer and centrifuged at 300 × g for 5 min. After supernatant removal, the pelleted cells were suspended in red blood cell lysis buffer (Miltenyi Biotec) to lyse the red blood cells. The cell pellets were subsequently washed, resuspended in PBS containing 0.04% BSA, and re‐filtered through a 35‐µm cell strainer. Dissociated single cells were stained with AM/PI for viability assessment using a Countstar Fluorescence Cell Analyzer.

### scRNA‐seq and TCR‐seq

The scRNA‐seq libraries and V(D)J libraries were obtained using the 10× Genomics Chromium Controller Instrument and Chromium Single Cell 5′ Library & Gel Bead kit, together with the V(D)J Enrichment Kit (10× Genomics). After concentration to 1000 cells/µL, the cells were loaded into each channel to generate single‐cell gel bead‐in‐emulsions (GEMs), which resulted in the expected mRNA barcoding of 5000 single cells for each sample. The GEMs were broken and barcoded complementary (c)‐DNA was purified and amplified after the reverse transcription step. The amplified barcoded cDNA was fragmented, A‐tailed, ligated with adaptors, and amplified by polymerase chain reaction (PCR), which ultimately generated 5′ gene expression libraries. For the V(D)J library, human T cell V(D)J sequences were enriched from the amplified cDNA, followed by fragmentation, A‐tailing, adaptor ligation and index PCR amplification. The final libraries were quantified using the Qubit High Sensitivity DNA assay (Thermo Fisher Scientific), and the size distribution of the libraries was determined using a High Sensitivity DNA chip on a Bioanalyzer 2200 (Agilent). All libraries were analyzed using an Illumina sequencer (Illumina) with 150‐bp paired‐end reads.

### scRNA‐seq Data Processing and Analysis

Sequencing data analysis was performed by NovelBio Co., Ltd. using the NovelBrain Cloud Analysis Platform (www.novelbrain.com). Fastp^[^ ^47^ ^]^ with default parameter filtering of the adaptor sequence was applied, and the low‐quality reads were removed. Then, feature‐barcode matrices were obtained by aligning reads to the human genome (GRCh38 Ensemble: version 100) using CellRanger v5.0.1. Next, downsampling analysis among samples sequenced according to the mapped barcoded reads per cell of each sample was applied and finally an aggregated matrix was achieved. Low‐quality cells were discarded if the number of expressed genes was <200 or if the mitochondrial UMI rate was >20%. The mitochondrial genes were removed from the expression table. Further analysis was conducted using the Seurat package (version 3.1.4 https://satijalab.org/seurat/). Based on the expression table according to the UMI counts of each sample and percent of mitochondria rate, raw feature counts were log‐normalized, scaled and subjected to principal component analysis (PCA), which was performed based on the scaled data of the top 2000 highly variable genes. The top 10 PCs were used for tSNE and UMAP construction, and unsupervised cell cluster results based on the top 10 PCs were obtained using a graph‐based cluster method. The marker genes using the FindAllMarkers function with the Wilcoxon rank sum test algorithm were calculated with the following criteria: 1) lnFC > 0.25; 2) *p*‐value <0.05; and 3) min.pct > 0.1. To identify specific cell type, clusters of the same cell type were selected for re‐tSNE/re‐UMAP analysis, graph‐based clustering, and marker analysis.

### CNV Estimation

Cells defined as endothelial cells, fibroblasts and smooth muscle cells were used as references to identify somatic CNVs with the R package inferCNV (version 0.8.2). Each cell was scored according to the extent of the CNV signal, defined as the mean of the squares of the CNV values across the genome. Putative malignant cells were defined as those with a CNV signal >0.05 and CNV correlation >0.5.

### HPV Sequence Blasting Analysis

Common high‐risk HPV gene sequences, including HPV 16, 18, 58, 52, 31 and 45 are shown in Table S4, Supporting Information.

### Differential Gene Expression Analysis

To identify DEGs among groups (malignant epithelial cells versus [versus] NMECs; HPV infected epithelial cells versus non‐HPV infected epithelial cells; and SSECs versus GECs), the function FindMarkers with Wilcoxon rank sum test algorithm was used. Significantly DEGs were selected as those meeting the following criteria: (1) lnFC > 0.25, 2) *p‐value* < 0.05, and 3) min.pct > 0.1.

### GO Analysis

GO^[^ ^48^ ^]^ analysis was performed to elucidate the biological implications of DEGs and marker genes. GO annotations were downloaded from the NCBI (http://www.ncbi.nlm.nih.gov/), GO (http://www.geneontology.org/), and UniProt (http://www.UniProt.org/) databases. The Fisher exact test was used to identify the significant GO categories, and a false discovery rate (FDR) was used to correct the *p*‐values.

### Pathway Analysis

Pathway analysis was used to explore the significant pathways of the DEGs and marker genes based on the Kyoto Encyclopedia of Genes and Genomes (KEGG) database. The Fisher exact test was used to identify significant pathways, and the threshold of significance was defined using the *p*‐value and false discovery rate (FDR).

### QuSAGE Analysis

To characterize the relative activation of a given gene set, such as the KEGG, Hallmark (h.all.v7.0.symbols, https://www.gsea‐msigdb.org/gsea/msigdb/index.jsp), and Reactome (https://reactome.org) gene sets QuSAGE (2.16.1) analysis was performed.^[^ ^49^ ^]^

### SCENIC Analysis

To assess the regulatory strength of TFs, the SCENIC (version 0.9.5) workflow was used,^[^ ^50^ ^]^ which is a new computational method for the construction of regulatory networks and identification of different cell states from scRNA‐seq data, using the 20 000 motif database for RcisTarget and GRNboost.

### Pseudotime Analysis

Single‐cell trajectory analysis was performed using Monocle2^[^ ^51^ ^]^ (http://cole‐trapnell‐lab.github.io/monocle‐release) with DDR‐Tree and default parameters to determine the dramatic translational relationships among cell types and clusters. Before performing Monocle analysis, marker genes of the Seurat clustering result and raw expression counts of the cells that passed filtering were selected.

### CytoTRACE

CytoTRACE,^[^ ^52^ ^]^ a computational method, was used to predict the differentiation state of cells from scRNA‐seq data by counting the number of detectably expressed genes per cell.

### Cell–Cell Communication Analysis

Cellular interaction analysis was conducted with CellPhoneDB (version 1.1.0),^[^ ^53^ ^]^ a public repository of ligands, receptors and their interactions, which enables systematic analysis of cell–cell communication at the molecular level. The membrane, secreted and peripheral proteins were annotated. Based on the interaction and the normalized cell matrix achieved by Seurat normalization, the rank and mean significance were calculated (*p‐value* < 0.05).

### Single‐Cell Gene Set Enrichment Analysis

Single‐cell gene set enrichment analysis (ssGSEA) was performed based on the domestic gene set and normalized gene expression matrix by single‐cell gene set enrichment analysis (ssGSEA) function in the gene set variation analysis (GSVA) package^[^ ^54^ ^]^ to obtain the gene enrichment score of each cell, including gene sets from the KEGG pathway database (https://www.kegg.jp/) and MSigDB (http://www.gsea‐msigdb.org/gsea/msigdb/index.jsp).

### TCGA Public Dataset Analysis

RNA‐seq data, clinicopathological information, and survival data of the CESC dataset from the TCGA database were downloaded from Xena (https://xena.ucsc.edu/). Survival analysis was conducted using the powerful online tool GEPIA (http://gepia.cancer‐pku.cn/)^[^ ^55^ ^]^ or R software (version 3.6.2; R Foundation for Statistical Computing) with packages including survival and rms. The RNA‐seq data included 257 SCC and 32 ADC samples, with the expression of each gene using the log2(TPM + 1) scale.

### IHC Analysis

To determine the proportion of epithelial cells, endothelial cells and fibroblasts in the CC TME, one SCC sample and one ADC sample were fixed in 4% paraformaldehyde (Servicebio) for paraffin embedding. Thereafter, 4‐µm‐thick sections were prepared. The following antibodies and dilutions were used to detect proteins: anti‐pan‐keratin (mouse, 1:250, 4545, Cell Signaling Technology), anti‐vimentin (rabbit, 1:100, 5741, Cell Signaling Technology), and anti‐CD31 (rabbit, 1:200, 77699, Cell Signaling Technology). According to the percentage of positive cells, the IHC staining area score was divided as follows: 0 point (<5%), 1 point (5–25%), 2 points (26–50%), 3 points (51–75%) %), and 4 points (76–100%), whereas the IHC staining intensity was divided as follows: 0 point (negative); 1 point (weak positive); 2 points (positive); 3 points (strong positive). Immunoreactive scores were calculated by multiplying the area and intensity scores.

### Organoids Dynamic Observation

To distinguish and track the different cell types, tumor epithelial cells (SiHa and HeLa) and HUVECs were transfected with lentiviral vectors carrying a cytomegalovirus promoter driving the expression of green (GFP, GeneChem) and red (mCherry, GeneChem) fluorescent proteins. After puromycin selection (1 µg mL^−1^, Thermo Fisher Scientific), SiHa/HeLa cells stably expressing GFP and HUVECs stably expressing mCherry were used for further organoid generation and screening. To test invasive ability, the organoids with SiHa‐GFP/HeLa‐GFP, HUVEC‐mCherry and DAPI‐stained CAFs were generated and observed under a fluorescence microscope to record their morphologic shapes.

### qRT–PCR

The primer sequences used for amplification of *TP63*, *KRT14*, *TP63*, *S100A8*, *CLDN3*, *MMP2*, *RHOA*, *ROCK1*, *CDC42*, and glyceraldehyde‐3‐phosphate dehydrogenase (*GAPDH*) are listed in Table S5, Supporting Information. The relative expression of each gene was calculated based on the corresponding Ct values, which were normalized to the *GAPDH* expression. Fold changes in the expression of each gene were calculated using the comparative Ct method with the 2^−ΔΔCt^ relative quantification method.

### Animal Experiments

All experiments were performed under the approval of Fudan University Animal Care and Use Committee. Female C57BL/6 mice (5–7 weeks old) were purchased from the Laboratory Animal Center of the Shanghai Institutes for Biological Sciences and were housed in a pathogen‐free environment. The TC‐1 cell line was purchased from the Chinese Academy of Sciences Shanghai Cellular Library. The cells were maintained in the RPMI 1640 medium (Thermo Fisher Scientific) supplemented with 100 IU/mL penicillin G, 100 mg mL^−1^ of streptomycin sulfate, and 10% of FBS (Thermo Fisher Scientific). To establish ectopic tumors, 1 × 10^6^ TC‐1 cells (in 200 µl of PBS) were injected subcutaneously into the right shoulders of C57BL/6 mice. When the tumor size reached ≈50 mm^3^, the tumor‐bearing mice were randomly divided into four groups (*n* = 6 for each group): the control, anti‐PD1, anti‐TIGIT, and anti‐CD96 groups (denoted as Day 0). For PD‐1, CD96 and TIGIT blockades, transplant recipients received 200 µg of anti‐PD‐1 (Bioxcell, BE0146), 200 µg of anti‐CD96 (Bioxcell, BE0337) and 200 µg of anti‐TIGIT (BE0274) twice a week × 4 doses (Day 3, Day 6, Day9, Day 12) through intraperitoneal injection. For the control group, the mice received an immunoglobulin G control (BioLegend) at a dose of 200 µg/mouse on a similar schedule. For immune response analysis, the tumors were excised on day 12. Tumor tissues were digested in the RPMI 1640 medium supplemented with 0.2% collagenase type IV, 0.01% hyaluronidase, and 0.002% DNase I for 3 h at 37 °C in 5% carbon dioxide. The TILs were separated using Ficoll (Dakewe Biotech Company), followed by flow cytometry analysis. The tumor size was monitored every 3 days and measured at the time of sacrifice.

### Flow Cytometry

To detect intracellular proteins, cells were stimulated for 6 h with 10 µg mL^−1^ brefeldin A and 2 µm of ionomycin (Absin) before staining. Then, the cells were fixed and permeabilized using a fixation/permeabilization wash buffer (BioLegend) and stained with antibodies for 30 min in the dark. For Ki67 staining, cells were incubated in 70% ethanol for 1 h at −20 °C and then stained with an anti‐Ki67 antibody (563757; BD Biosciences). All the antibodies used in this study are listed in Table S6, Supporting Information. All samples were run on a CytoFLEX platform (Beckman Coulter) and analyzed using FlowJo version 10.8 software (BD Biosciences).

### Quantification and Statistical Analysis

Specific statistical tests and metrics (mean value ± standard deviation or median value with interquartile range) used for comparisons along with sample sizes were described in the results and figure legends sections. Comparisons of continuous data of different data types between two groups were performed using the unpaired two‐tailed Student *t*‐test, Mann–Whitney test or Kruskal–Wallis test; one‐way analysis of variance with the Tukey post hoc test was used to compare data between multiple groups. Kaplan–Meier curves between the two groups were analyzed using the log‐rank test. All statistical analyses were performed with GraphPad Prism (version 8, GraphPad Software) or R (version 3.6.2). Statistical significance was set at *p* < 0.05.

## Conflict of Interest

The authors declare no conflict of interest.

## Author Contributions

J.Q., X.Q., and Y.W. contributed equally to this work. J.Q., X.Q., and L.W. developed the concept and discussed experiments. X.Q. and Y.W. collected patient samples and data. X.Q., C.G., and J.Q. contributed to bioinformatic data analysis and interpretation. X.Q., Y.W., L.W., and W.S. designed, performed and analyzed experiments. J.Q., X.Q., L.W., and K.H. wrote and edited the manuscript. B.L. and X.Q. contributed to graph drawing. J.Q. and K.H. supervised the progress of the study.

## Supporting information

Supporting Information

## Data Availability

The data that support the findings of this study are available from the corresponding author upon reasonable request.
