# Supplementary material for: Single‐Cell Landscape Highlights Heterogenous Microenvironment, Novel Immune Reaction Patterns, Potential Biomarkers and Unique Therapeutic Strategies of Cervical Squamous Carcinoma, Human Papillomavirus‐Associated (HPVA) and Non‐HPVA Adenocarcinoma
Source: Adv Sci (Weinh). 2023 Feb 1;10(10):2204951. doi: 10.1002/advs.202204951 (PMC10074047; doi:10.1002/advs.202204951)
Supplement: Supplementary file 1 — Supporting Information [file ADVS-10-2204951-s001.pdf]

ADVANCED SCIENCE
Open Access

Supporting Information

for *Adv. Sci.*, DOI 10.1002/advs.202204951

Single-Cell Landscape Highlights Heterogenous Microenvironment, Novel Immune Reaction Patterns, Potential Biomarkers and Unique Therapeutic Strategies of Cervical Squamous Carcinoma, Human Papillomavirus-
Associated (HPVA) and Non-HPVA Adenocarcinoma

Junjun Qiu, Xinyu Qu, Yumeng Wang, Chenyan Guo, Bin Lv, Qian Jiang, Wentao Su, Li Wang* and Keqin Hua**

Supporting information

Single-cell landscape highlights heterogenous microenvironment, novel immune reaction patterns, potential biomarkers and unique therapeutic strategies of cervical squamous carcinoma, human papillomavirus-associated (HPVA) and non-HPVA adenocarcinoma

Junjun Qiu[1,2#], Xinyu Qu[1,2#], Yumeng Wang[1,2#], Chenyan Guo[1,2], Bin Lv[1,2], Qian Jiang[1,2], Wentao Su[5], Li Wang[3,4*], Keqin Hua[1,2*]*

Table S1

Clinical characteristics of each sample and numbers of cells sequenced after quality control procedures

Sample	Age	Pathological Diagnosis	Figo Stage	HPV Infection	Number of cells
SCC_1	52	squamous cell carcinoma	IIA1	HPV58+	2601
SCC_2	63	squamous cell carcinoma	IIA1	HPV16+	6747
SCC_3	37	squamous cell carcinoma	IB2	HPV16+	5389
ADC_1	57	adenocarcinoma of cervix, usual type	IB2	HPV16+	3974
ADC_2	27	adenocarcinoma of cervix, usual type	IB1	HPV18+	1865
ADC_3	43	adenocarcinoma of cervix, mucinous type	IB2	HPV45+	2172
ADC_4	48	NHPVA, endometrioid type	IB3	-	3815
ADC_5	43	NHPVA, gastric type	IIA1	-	3095

Table S2

Pathologic characteristics of the 15 cervical cancer samples used for IHC staining.

Sample	Pathologic Diagnosis	TNM Stage	Sample	Pathologic Diagnosis	TNM Stage
FFPE_SCC_1	squamous cell carcinoma	T1b2N1M0	FFPE_SCC_9	squamous cell carcinoma	T1b1N0M0
FFPE_SCC_2	squamous cell carcinoma	T2a1N0M0	FFPE_SCC_10	squamous cell carcinoma	T2a2N1M0
FFPE_SCC_3	squamous cell carcinoma	T2a2N0M0	FFPE_ADC_1	adenocarcinoma	T1b1N0M0
FFPE_SCC_4	squamous cell carcinoma	T2a2N0M0	FFPE_ADC_2	adenocarcinoma	T2a2N1M0
FFPE_SCC_5	squamous cell carcinoma	T1b1N0M0	FFPE_ADC_3	adenocarcinoma	T1b1N0M0
FFPE_SCC_6	squamous cell carcinoma	T1b1N0M0	FFPE_ADC_4	adenocarcinoma	T1b2N0M0
FFPE_SCC_7	squamous cell carcinoma	T2bN1M0	FFPE_ADC_5	adenocarcinoma	T1b1N0M0
FFPE_SCC_8	squamous cell carcinoma	T1b1N0M0			

Table S3

Pathologic characteristics of the 20 cervical cancer samples used for qRT-PCR.

**Sample**	**Pathologic Diagnosis**	**TNM Stage**	**Sample**	**Pathologic Diagnosis**	**TNM Stage**
Frozen_SCC_1	squamous cell carcinoma	T2bN1M0	Frozen_ADC_1	adenocarcinoma	T1b2N0M0
Frozen_SCC_2	squamous cell carcinoma	T2a1N0M0	Frozen_ADC_2	adenocarcinoma	T2a2N1M0
Frozen_SCC_3	squamous cell carcinoma	T1b1N0M0	Frozen_ADC_3	adenocarcinoma	T1b1N0M0
Frozen_SCC_4	squamous cell carcinoma	T1b2N1M0	Frozen_ADC_4	adenocarcinoma	T1b2N1M0
Frozen_SCC_5	squamous cell carcinoma	T1b2N0M0	Frozen_ADC_5	adenocarcinoma	T1b2N0M0
Frozen_SCC_6	squamous cell carcinoma	T2a1N1M0	Frozen_ADC_6	adenocarcinoma	T2a1N1M0
Frozen_SCC_7	squamous cell carcinoma	T1b2N1M0	Frozen_ADC_7	adenocarcinoma	T1b1N0M0
Frozen_SCC_8	squamous cell carcinoma	T2a1N0M0	Frozen_ADC_8	adenocarcinoma	T1b2N1M0
Frozen_SCC_9	squamous cell carcinoma	T1b2N0M0	Frozen_ADC_9	adenocarcinoma	T1b2N0M0
Frozen_SCC_10	squamous cell carcinoma	T1b2N1M0	Frozen_ADC_10	adenocarcinoma	T2a1N1M0

Table S4

Common High risk (Hr) HPV gene sequences including HPV 16, 18, 58, 52, 31 and 45

HPV subtype	Gene sequence
HPV 16	1atggctgatc ctgcaggtac caatggggaa gagggtacgg gatgtaatgg atggttttat
	61 gtagaggctg tagtggaaaa aaaaacaggg gatgctatat cagatgacga gaacgaaaat
	121 gacagtgata caggtgaaga tttggtagat tttatagtaa atgataatga ttatttaaca
	181 caggcagaaa cagagacagc acatgcgttg tttactgcac aggaagcaaa acaacataga
	241 gatgcagtac aggttctaaa acgaaagtat ttgggtagtc cacttagtga tattagtgga
	301 tgtgtagaca ataatattag tcctagatta aaagctatat gtatagaaaa acaaagtaga
	361 gctgcaaaaa ggagattatt tgaaagcgaa gacagcgggt atggcaatac tgaagtggaa
	421 actcagcaga tgttacaggt agaagggcgc catgagactg aaacaccatg tagtcagtat
	481 agtggtggaa gtgggggtgg ttgcagtcag tacagtagtg gaagtggggg agagggtgtt
	541 agtgaaagac acactatatg ccaaacacca cttacaaata ttttaaatgt actaaaaact
	601 agtaatgcaa aggcagcaat gttagcaaaa tttaaagagt tatacggggt gagtttttca
	661 gaattagtaa gaccatttaa aagtaataaa tcaacgtgtt gcgattggtg tattgctgca
	721 tttggactta cacccagtat agctgacagt ataaaaacac tattacaaca atattgttta
	781 tatttacaca ttcaaagttt agcatgttca tggggaatgg ttgtgttact attagtaaga
	841 tataaatgtg gaaaaaatag agaaacaatt gaaaaattgc tgtctaaact attatgtgtg
	901 tctccaatgt gtatgatgat agagcctcca aaattgcgta gtacagcagc agcattatat
	961 tggtataaaa caggtatatc aaatattagt gaagtgtatg gagacacgcc agaatggata
	1021 caaagacaaa cagtattaca acatagtttt aatgattgta catttgaatt atcacagatg
	1081 gtacaatggg cctacgataa tgacatagta gacgatagtg aaattgcata taaatatgca
	1141 caattggcag acactaatag taatgcaagt gcctttctaa aaagtaattc acaggcaaaa
	1201 attgtaaagg attgtgcaac aatgtgtaga cattataaac gagcagaaaa aaaacaaatg
	1261 agtatgagtc aatggataaa atatagatgt gatagggtag atgatggagg tgattggaag
	1321 caaattgtta tgtttttaag gtatcaaggt gtagagttta tgtcattttt aactgcatta
	1381 aaaagatttt tgcaaggcat acctaaaaaa aattgcatat tactatatgg tgcagctaac
	1441 acaggtaaat cattatttgg tatgagttta atgaaatttc tgcaagggtc tgtaatatgt
	1501 tttgtaaatt ctaaaagcca tttttggtta caaccattag cagatgccaa aataggtatg
	1561 ttagatgatg ctacagtgcc ctgttggaac tacatagatg acaatttaag aaatgcattg
	1621 gatggaaatt tagtttctat ggatgtaaag catagaccat tggtacaact aaaatgccct
	1681 ccattattaa ttacatctaa cattaatgct ggtacagatt ctaggtggcc ttatttacat
	1741 aatagattgg tggtgtttac atttcctaat gagtttccat ttgacgaaaa cggaaatcca
	1801 gtgtatgagc ttaatgataa gaactggaaa tcctttttct caaggacgtg gtccagatta
	1861 agtttgcacg aggacgagga caaggaaaac gatggagact ctttgccaac gtttaaatgt
	1921 gtgtcaggac aaaatactaa cacattatga aaatgatagt acagacctac gtgaccatat
	1981 agactattgg aaacacatgc gcctagaatg tgctatttat tacaaggcca gagaaatggg
	2041 atttaaacat attaaccacc aggtggtgcc aacactggct gtatcaaaga ataaagcatt
	2101 acaagcaatt gaactgcaac taacgttaga aacaatatat aactcacaat atagtaatga
	2161 aaagtggaca ttacaagacg ttagccttga agtgtattta actgcaccaa caggatgtat
	2221 aaaaaaacat ggatatacag tggaagtgca gtttgatgga gacatatgca atacaatgca
	2281 ttatacaaac tggacacata tatatatttg tgaagaagca tcagtaactg tggtagaggg
	2341 tcaagttgac tattatggtt tatattatgt tcatgaagga atacgaacat attttgtgca
	2401 gtttaaagat gatgcagaaa aatatagtaa aaataaagta tgggaagttc atgcgggtgg
	2461 tcaggtaata ttatgtccta catctgtgtt tagcagcaac gaagtatcct ctcctgaaat
	2521 tattaggcag cacttggcca accaccccgc cgcgacccat accaaagccg tcgccttggg
	2581 caccgaagaa acacagacga ctatccagcg accaagatca gagccagaca ccggaaaccc
	2641 ctgccacacc actaagttgt tgcacagaga ctcagtggac agtgctccaa tcctcactgc
	2701 atttaacagc tcacacaaag gacggattaa ctgtaatagt aacactacac ccatagtaca
	2761 tttaaaaggt gatgctaata ctttaaaatg tttaagatat agatttaaaa agcattgtac
	2821 attgtatact gcagtgtcgt ctacatggca ttggacagga cataatgtaa aacataaaag
	2881 tgcaattgtt acacttacat atgatagtga atggcaacgt gaccaatttt tgtctcaagt
	2941 taaaatacca aaaactatta cagtgtctac tggatttatg tctatatgac aaatcttgat
	3001 actgcatcca caacattact ggcgtgcttt ttgctttgct tttgtgtgct tttgtgtgtc
	3061 tgcctattaa tacgtccgct gcttttgtct gtgtctacat acacatcatt aataatattg
	3121 gtattactat tgtggataac agcagcctct gcgtttaggt gttttattgt atatattata
	3181 tttgtttata taccattatt tttaatacat acacatgcac gctttttaat tacataatgt
	3241 atatgtacat aatgtaattg ttacatataa ttgttgtata ccataactta ctatttttc

3301 tttttattt tcatatataa ttttttttt tgtttgtttg tttgtttttt aataaactgt
3361 tattacttaa caatgcgaca caaacgttct gcaaaacgca caaaacgtgc atcggctacc
3421 caactttata aaacatgcaa acaggcaggt acatgtccac ctgacattat acctaaggtt
3481 gaaggcaaaa ctattgctga tcaaatatta caatatggaa gtatgggtgt attttttggt
3541 gggttaggaa ttggaacagg gtcgggtaca ggcggacgca ctgggtatat tccattggga
3601 acaaggcctc ccacagctac agatacactt gctcctgtaa gacccccttt aacagtagat
3661 cctgtgggcc cttctgatcc ttctatagtt tctttagtgg aagaaactag ttttattgat
3721 gctggtgcac caacatctgt accttccatt cccccagatg tatcaggatt tagtattact
3781 acttcaactg ataccacacc tgctatatta gatattaata atactgttac tactgttact
3841 acacataata atcccacttt cactgaccca tctgtattgc agcctccaac acctgcagaa
3901 actggagggc attttacact ttcatcatcc actattagta cacataatta tgaagaaatt
3961 cctatggata catttattgt tagcacaaac cctaacacag taactagtag cacacccata
4021 ccagggtctc gcccagtggc acgcctagga ttatatagtc gcacaacaca acaggttaaa
4081 gttgtagacc ctgcttttgt aaccactccc actaaactta ttacatatga taatcctgca
4141 tatgaaggta tagatgtgga taatacatta tatttttcta gtaatgataa tagtattaat
4201 atagctccag atcctgactt tttggatata gttgctttac ataggccagc attaacctct
4261 aggcgtactg gcattaggta cagtagaatt ggtaataaac aaacactacg tactcgtagt
4321 ggaaaatcta taggtgctaa ggtacattat tattatgatt taagtactat tgatcctgca
4381 gaagaaatag aattacaaac tataacacct tctacatata ctaccacttc acatgcagcc
4441 tcacctactt ctattaataa tggattatat gatatttatg cagatgactt tattacagat
4501 acttctacaa ccccggtacc atctgtaccc tctacatctt tatcaggtta tattcctgca
4561 aatacaacaa ttccttttgg tggtgcatac aatattcctt tagtatcagg tcctgatata
4621 cccattaata taactgacca agctccttca ttaattccta tagttccagg gtctccacaa
4681 tatacaatta ttgctgatgc aggtgacttt tatttacatc ctagttatta catgttacga
4741 aaacgacgta aacgtttacc atattttttt tcagatgtct ctttggctgc ctagtgaggc
4801 cactgtctac ttgcctcctg tcccagtatc taaggttgta agcacggatg aatatgttgc
4861 acgcacaaac atatattatc atgcaggaac atccagacta cttgcagttg gacatcccta
4921 ttttcctatt aaaaaaccta acaataacaa aatattagtt cctaaagtat caggattaca
4981 atacagggta tttagaatac atttacctga ccccaataag tttggttttc ctgacacctc
5041 attttataat ccagatacac agcggctggt ttgggcctgt gtaggtgttg aggtaggtcg
5101 tggtcagcca ttaggtgtgg gcattagtgg ccatccttta ttaaataaat tggatgacac
5161 agaaaatgct agtgcttatg cagcaaatgc aggtgtggat aatagagaat gtatatctat
5221 ggattacaaa caaacacaat tgtgtttaat tggttgcaaa ccacctatag ggaacactg
5281 gggcaaagga tccccatgta ccaatgttgc agtaaatcca ggtgattgtc caccattaga
5341 gttaataaac acagttattc aggatggtga tatggttgat actggctttg gtgctatgga
5401 ctttactaca ttacaggcta acaaaagtga agttccactg gatatttgta catctatttg
5461 caaatatcca gattatatta aaatggtgtc agaaccatat ggcgacagct tattttttta
5521 tttacgaagg gaacaaatgt ttgttagaca tttatttaat agggctggta ctgttggtga
5581 aaatgtacca gacgatttat acattaaagg ctctgggtct actgcaaatt tagccagttc
5641 aaattatttt cctacaccta gtggttctat ggttacctct gatgcccaaa tattcaataa
5701 accttattgg ttacaacgag cacagggcca caataatggc atttgttggg gtaaccaact
5761 atttgttact gttgttgata ctacacgcag tacaaatatg tcattatgtg ctgccatatc
5821 tacttcagaa actacatata aaaatactaa ctttaaggag tacctacgac atggggagga
5881 atatgattta cagtttattt ttcaactgtg caaaataacc ttaactgcag acgttatgac
5941 atacatacat tctatgaatt ccactatttt ggaggactgg aattttggtc tacaacctcc
6001 cccaggaggc acactagaag atacttatag gtttgtaaca tcccaggcaa ttgcttgtca
6061 aaaacataca cctccagcac ctaaagaaga tccccttaaa aaatacactt tttgggaagt
6121 aaatttaaag gaaaagtttt ctgcagacct agatcagttt cctttaggac gcaaattttt
6181 actacaagca ggattgaagg ccaaaccaaa atttacatta ggaaaacgaa aagctacacc
6241 caccacctca tctacctcta caactgctaa acgcaaaaaa cgtaagctgt aagtattgta
6301 tgtatgttga attagtgttg tttgttgtgt atatgtttgt atgtgcttgt atgtgcttgt
6361 aaatattaag ttgtatgtgt gtttgtatgt atggtataat aaacacgtgt gtatgtgttt
6421 ttaaatgctt gtgtaactat tgtgtcatgc aacataaata aacttattgt ttcaacacct
6481 actaattgtg ttgtggttat tcattgtata taaactatat ttgctacatc ctgtttttgt
6541 tttatatata ctatattttg tagcgccagc ggccattttg tagcttcaac cgaattcggt
6601 tgcatgcttt ttggcacaaa atgtgttttt ttaaatagtt ctatgtcagc aactatggtt
6661 taaacttgta cgtttcctgc ttgccatgcg tgccaaatcc ctgttttcct gacctgcact

	6721 gcttgccaac cattccattg ttttttacac tgcactatgt gcaactactg aatcactatg
	6781 tacattgtgt catataaaat aaatcactat gcgccaacgc cttacatacc gctgttaggc
	6841 acatattttt ggcttgtttt aactaaccta attgcatatt tggcataagg tttaaacttc
	6901 taaggccaac taaatgtcac cctagttcat acatgaactg tgtaaaggtt agtcatacat
	6961 tgttcatttg taaaactgca catgggtgtg tgcaaaccgt tttgggttac acatttacaa
	7021 gcaacttata taataatact aaactacaat aattcatgta taaaactaag ggcgtaaccg
	7081 aaatcggttg aaccgaaacc ggttagtata aaagcagaca ttttatgcac caaaagagaa
	7141 ctgcaatgtt tcaggaccca caggagcgac ccagaaagtt accacagtta tgcacagagc
	7201 tgcaaacaac tatacatgat ataatattag aatgtgtgta ctgcaagcaa cagttactgc
	7261 gacgtgaggt atatgacttt gcttttcggg atttatgcat agtatataga gatgggaatc
	7321 catatgctgt atgtgataaa tgtttaaagt tttattctaa aattagtgag tatagacatt
	7381 attgttatag tttgtatgga acaacattag aacagcaata caacaaaccg ttgtgtgatt
	7441 tgttaattag gtgtattaac tgtcaaaagc cactgtgtcc tgaagaaaag caaagacatc
	7501 tggacaaaaa gcaaagattc cataatataa ggggtcggtg gaccggtcga tgtatgtctt
	7561 gttgcagatc atcaagaaca cgtagagaaa cccagctgta atcatgcatg gagatacacc
	7621 tacattgcat gaatatatgt tagatttgca accagagaca actgatctct actgttatga
	7681 gcaattaaat gacagctcag aggaggagga tgaaatagat ggtccagctg gacaagcaga
	7741 accggacaga gcccattaca atattgtaac cttttgttgc aagtgtgact ctacgcttcg
	7801 gttgtgcgta caaagcacac acgtagacat tcgtactttg gaagacctgt taatgggcac
	7861 actaggaatt gtgtgcccca tctgttctca gaaaccataa tctacc
HPV18	1 attaatactt ttaacaattg tagtatataa aaaagggagt aaccgaaaac ggtcgggacc
	61 gaaaacggtg tatataaaag atgtgagaaa cacaccacaa tactatggcg cgctttgagg
	121 atccaacacg gcgaccctac aagctacctg atctgtgcac ggaactgaac acttcactgc
	181 aagacataga aataacctgt gtatattgca agacagtatt ggaacttaca gaggtatttg
	241 aatttgcatt taaagattta tttgtggtgt atagagacag tataccgcat gctgcatgcc
	301 ataaatgtat agatttttat tctagaatta gagaattaag acattattca gactctgtgt
	361 atggagacac attggaaaaa ctaactaaca ctgggttata caatttatta ataaggtgcc
	421 tgcggtgcca gaaaccgttg aatccagcag aaaaacttag acaccttaat gaaaaacgac
	481 gatttcacaa catagctggg cactatagag gccagtgcca ttcgtgctgc aaccgagcac
	541 gacaggaacg actccaacga cgcagagaaa cacaagtata atattaagta tgcatggacc
	601 taaggcaaca ttgcaagaca ttgtattgca tttagagccc caaaatgaaa ttccggttga
	661 ccttctatgt cacgagcaat taagcgactc agaggaagaa aacgatgaaa tagatggagt
	721 taatcatcaa catttaccag cccgacgagc cgaaccacaa cgtcacacaa tgttgtgtat
	781 gtgttgtaag tgtgaagcca gaattgagct agtagtagaa agctcagcag acgaccttcg
	841 agcattccag cagctgtttc tgaacaccct gtcctttgtg tgtccgtggt gtgcatccca
	901 gcagtaagca acaatggctg atccagaagg tacagacggg gagggcacgg gttgtaacgg
	961 ctggttttat gtacaagcta ttgtagacaa aaaaacagga gatgtaatat cagatgacga
	1021 ggacgaaaat gcaacagaca cagggtcgga tatggtagat tttattgata cacaaggaac
	1081 attttgtgaa caggcagagc tagagacagc acaggcattg ttccatgcgc aggaggtcca
	1141 caatgatgca caagtgttgc atgttttaaa acgaaagttt gcaggaggca gcaaagaaaa
	1201 cagtccatta ggggagcggc tggaggtgga tacagagtta agtccacggt tacaagaaat
	1261 atctttaaat agtgggcaga aaaaggcaaa aaggcggctg tttacaatat cagatagtgg
	1321 ctatggctgt tctgaagtgg aagcaacaca gattcaggta actacaaatg gcgaacatgg
	1381 cggcaatgta tgtagtggcg gcagtacgga ggctatagac aacgggggca cagagggcaa
	1441 caacagcagt gtagacggta caagtgacaa tagcaatata gaaaatgtaa atccacaatg
	1501 taccatagca caattaaaag acttgttaaa agtaaacaat aaacaaggag ctatgttagc
	1561 agtatttaaa gacacatatg ggctatcatt tacagattta gttagaaatt ttaaaagtga
	1621 taaaaccacg tgtacagatt gggttacagc tatatttgga gtaaacccaa caatagcaga
	1681 aggatttaaa acactaatac agccatttat attatatgcc catattcaat gtctagactg
	1741 taaatgggga gtattaatat tagccctgtt gcgttacaaa tgtggtaaga gtagactaac
	1801 agttgctaaa ggtttaagta cgttgttaca cgtacctgaa acttgtatgt taattcaacc
	1861 accaaaattg cgaagtagtg ttgcagcact atattggtat agaacaggaa tatcaaatat
	1921 tagtgaagta atgggagaca cacctgagtg gatacaaaga cttactatta tacaacatgg
	1981 aatagatgat agcaattttg atttgtcaga aatggtacaa tgggcatttg ataatgagct
	2041 gacagatgaa agcgatatgg catttgaata tgccttatta gcagacagca acagcaatgc
	2101 agctgccttt ttaaaaagca attgccaagc taaatattta aaagattgtg ccacaatgtg
	2161 caaacattat aggcgagccc aaaaacgaca aatgaatatg tcacagtgga tacgatttag

2221 atgttcaaaa atagatgaag ggggagattg gagaccaata gtgcaattcc tgcgatacca
2281 acaaatagag tttataacat ttttaggagc cttaaaatta tttttaaaag gaaccccca
2341 aaaaaattgt ttagtatttt gtggaccagc aaatacagga aaatcatatt ttggaatgag
2401 ttttatacac tttatacaag gagcagtaat atcatttgtg aattccacta gtcatttttg
2461 gttggaaccg ttaacagata ctaaggtggc catgttagat gatgcaacga ccacgtgttg
2521 gacatacttt gatacctata tgagaaatgc gttagatggc aatccaataa gtattgatag
2581 aaagcacaaa ccattaatac aactaaaatg tcctccaata ctactaacca caaatataca
2641 tccagcaaag gataatagat ggccatattt agaaagtaga ataacagtat ttgaatttcc
2701 aaatgcattt ccatttgata aaaatggcaa tccagtatat gaaataaatg acaaaaattg
2761 gaaatgtttt tttgaaagga catggtccag attagatttg cacgaggaag aggaagatgc
2821 agacaccgaa ggaaaccctt tcggaacgtt taagtgcgtt gcaggacaaa atcatagacc
2881 actatgaaaa tgacagtaaa gacatagaca gccaaataca gtattggcaa ctaatacgtt
2941 gggaaaatgc aatattcttt gcagcaaggg aacatggcat acagacatta aaccaccagg
3001 tggtgccagc ctataacatt tcaaaaagta aagcacataa agctattgaa ctgcaaatgg
3061 ccctacaagg ccttgcacaa agtgcataca aaaccgagga ttggacactg caagacacat
3121 gcgaggaact atggaataca gaacctactc actgctttaa aaaaggtggc caaacagtac
3181 aagtatattt tgatggcaac aaagacaatt gtatgaacta tgtagcatgg gacagtgtgt
3241 attatatgac tgatgcagga acatgggaca aaacggctac ctgtgtaagt cacaggggat
3301 tgtattatgt aaaggaaggg tacaacacgt tttatataga atttaaaagt gaatgtgaaa
3361 aatatgggaa cacaggtacg tgggaagtac attttgggaa taatgtaatt gattgtaatg
3421 actctatgtg cagtaccagt gacgacacgg tatccgctac tcagcttgtt aaacagctac
3481 agcacacccc ctcaccgtat tccagcaccg tgtccgtggg caccgcaaag acctacggcc
3541 agacgtcggc tgctacacga cctggacact gtggactcgc ggagaagcag cattgtggac
3601 ctgtcaaccc acttctcggt gcagctacac ctacaggcaa caacaaaaga cggaaactct
3661 gtagtggtaa cactacgcct ataatacatt taaaaggtga cagaaacagt ttaaaatgtt
3721 tacggtacag attgcgaaaa catagcgacc actatagaga tatatcatcc acctggcatt
3781 ggacaggtgc aggcaatgaa aaaacaggaa tactgactgt aacataccat agtgaaacac
3841 aaagaacaaa atttttaaat actgttgcaa ttccagatag tgtacaaata ttggtgggat
3901 acatgacaat gtaatacata tgctgtagta ccaatatgtt atcacttatt tttttatttt
3961 gcttttgtgt atgcatgtat gtgtgctgcc atgtcccgct tttgccatct gtctgtatgt
4021 gtgcgtatgc atgggtattg gtatttgtgt atattgtggt aataacgtcc cctgccacag
4081 cattcacagt atatgtattt tgttttttat tgcccatgtt actattgcat atacatgcta
4141 tattgtcttt acagtaattg tataggttgt tttatacagt gtattgtaca ttgtatattt
4201 tgttttatac cttttatgct ttttgtattt ttgtaataaa agtatggtat cccaccgtgc
4261 cgcacgacgc aaacgggctt cggtaactga cttatataaa acatgtaaac aatctggtac
4321 atgtccacct gatgttgttc ctaaggtgga gggcaccacg ttagcagata aaatattgca
4381 atggtcaagc cttggtatat ttttgggtgg acttggcata ggtactggca gtggtacagg
4441 gggtcgtaca gggtacattc cattgggtgg gcgttccaat acagtggtgg atgttggtcc
4501 tacacgtccc ccagtggtta ttgaacctgt gggccccaca gacccatcta ttgttacatt
4561 aatagaggac tccagtgtgg ttacatcagg tgcacctagg cctacgttta ctggcacgtc
4621 tgggtttgat ataacatctg cgggtacaac tacacctgcg gttttggata tcacaccttc
4681 gtctacctct gtgtctattt ccacaaccaa ttttaccaat cctgcatttt ctgatccgtc
4741 cattattgaa gttccacaaa ctggggaggt gtcaggtaat gtatttgttg gtacccctac
4801 atctggaaca catgggtatg aggaaatacc tttacaaaca tttgcttctt ctggtacagg
4861 ggaggaaccc attagtagta ccccattgcc tactgtgcgg cgtgtagcag gtccccgcct
4921 ttacagtagg gcctaccaac aagtgtcagt ggctaaccct gagtttctta cacgtccatc
4981 ctctttaatt acatatgaca acccggcctt tgagcctgtg gacactacat taacatttga
5041 tcctcgtagt gatgttcctg attcagattt tatggatatt atccgtctac ataggcctgc
5101 tttaacatcc aggcgtggga ctgttcgctt tagtagatta ggtcaaaggg caactatgtt
5161 tacccgcagc ggtacacaaa taggtgctag ggttcacttt tatcatgata taagtcctat
5221 tgcaccttcc ccagaatata ttgaactgca gcctttagta tctgccacgg aggacaatga
5281 cttgtttgat atatatgcag atgacatgga ccctgcagtg cctgtaccat cgcgttctac
5341 tacctccttt gcattttcta aatattcgcc cactatatct tctgcctctt cctatagtaa
5401 tgtaacggtc cctttaacct cctcttggga tgtgcctgta tacacgggtc ctgatattac
5461 attaccatct actacctctg tatggcccat tgtatcaccc acagcccctg cctctacaca
5521 gtatattggt atacatggta cacattatta tttgtggcca ttatattatt ttcttcctaa
5581 gaaacgtaaa cgtgttccct attttttgc agatggcttt gtggcggcct agtgacaata

	5641 ccgtatatct tccacctcct tctgtggcaa gagttgtaaa taccgatgat tatgtgactc
	5701 gcacaagcat attttatcat gctggcagct ctagattatt aactgttggt aatccatatt
	5761 ttagggttcc tgcaggtggt ggcaataagc aggatattcc taaggtttct gcataccaat
	5821 atagagtatt tagggtgcag ttacctgacc caaataaatt tggtttacct gatactagta
	5881 tttataatcc tgaaacacaa cgtttagtgt gggcctgtgc tggagtggaa attggccgtg
	5941 gtcagccttt aggtgttggc cttagtgggc atccatttta taataaatta gatgacactg
	6001 aaagttccca tgccgccacg tctaatgttt ctgaggacgt tagggacaat gtgtctgtag
	6061 attataagca gacacagtta tgtattttgg gctgtgcccc tgctattggg gaacactggg
	6121 ctaaaggcac tgcttgtaaa tcgcgtcctt tatcacaggg cgattgcccc cctttagaac
	6181 ttaaaaacac agttttggaa gatggtgata tggtagatac tggatatggt gccatggact
	6241 ttagtacatt gcaagatact aaatgtgagg taccattgga tatttgtcag tctatttgta
	6301 aatatcctga ttatttacaa atgtctgcag atccttatgg ggattccatg tttttttgct
	6361 tacggcgtga gcagcttttt gctaggcatt tttggaatag agcaggtact atgggtgaca
	6421 ctgtgcctca atccttatat attaaaggca caggtatgcg tgcttcacct ggcagctgtg
	6481 tgtattctcc ctctccaagt ggctctattg ttacctctga ctcccagttg tttaataaac
	6541 catattggtt acataaggca cagggtcata acaatggtgt ttgctggcat aatcaattat
	6601 ttgttactgt ggtagatacc actcgcagta ccaatttaac aatatgtgct tctacacagt
	6661 ctcctgtacc tggcaatat gatgctacca aatttaagca gtatagcaga catgttgagg
	6721 aatatgattt gcagtttatt tttcagttgt gtactattac tttaactgca gatgttatgt
	6781 cctatattca tagtatgaat agcagtattt tagaggattg gaactttggt gttccccccc
	6841 cgccaactac tagtttggtg gatacatatc gttttgtaca atctgttgct attacctgtc
	6901 aaaaggatgc tgcaccggct gaaaataagg atccctatga taagttaaag ttttggaatg
	6961 tggatttaaa ggaaaagttt tctttagact tagatcaata tcccccttgga cgtaaatttt
	7021 tggttcaggc tggattgcgt cgcaagccca ccataggccc tcgcaaacgt tctgctccat
	7081 ctgccactac gtcttctaaa cctgccaagc gtgtgcgtgt acgtgccagg aagtaatatg
	7141 tgtgtgtgta tatatatata catctattgt tgtgtttgta tgtcctgtgt ttgtgtttgt
	7201 tgtatgattg cattgtatgg tatgtatggt tgttgttgta tgttgtatgt tactatattt
	7261 gttggtatgt ggcattaaat aaaatatgtt ttgtggttct gtgtgttatg tggttgcgcc
	7321 ctagtgagta acaactgtat ttgtgtttgt ggtatgggtg ttgcttgttg ggctatatat
	7381 tgtcctgtat ttcaagttat aaaactgcac accttacagc atccatttta tcctacaatc
	7441 ctccattttg ctgtgcaacc gatttcggtt gcctttggct tatgtctgtg gttttctgca
	7501 caatacagta cgctggcact attgcaaact ttaatctttt gggcactgct cctacatatt
	7561 ttgaacaatt ggcgcgcctc tttggcgcat acaaggcgca cctggtatta gtcattttcc
	7621 tgtccaggtg cgctacaaca attgcttgca taactatatc cactccctaa gtaataaaac
	7681 tgcttttagg cacatatttt agtttgtttt tacttaagct aattgcatac ttggcttgta
	7741 caactacttt catgtccaac attctgtcta cccttaacat gaactataat atgactaagc
	7801 tgtgcataca tagtttatgc aaccgaaata ggttgggcag cacatactat acttttc
HPV58	1 ctaaactata atgccaaatc ttgtaaaaac tagggtgtaa ccgaaaacgg tctgaccgaa
	61 accggtgcat atataaagca gacatttttt ggtaggctac tgcaggacta tgttccagga
	121 cgcagaggag aaacccacgga cattgcatga tttgtgtcag gcgttggaga catctgtgca
	181 tgaaatcgaa ttgaaatgcg ttgaatgcaa aaagactttg cagcgatctg aggtatatga
	241 ctttgtattt gcagatttaa gaatagtgta tagagatgga aatccatttg cagtatgtaa
	301 agtgtgttta cgattgctat ctaaaataag tgagtataga cattataatt attcgctata
	361 tggagacaca ttagaacaaa cactaaaaaa gtgtttaaat gaaatattaa ttagatgtat
	421 tatttgtcaa agaccattgt gtccacaaga aaaaaaaagg catgtggatt taaacaaaag
	481 gtttcataat atttcgggtc gttggacagg gcgctgtgca gtgtgttgga gaccccgacg
	541 tagacaaaca caagtgtaac ctgtaacaac gccatgagag gaaacaaccc aacgctaaga
	601 gaatatattt tagatttaca tcctgaacca actgacctat tctgctatga gcaattatgt
	661 gacagctcag acgaggatga aataggcttg gacaggccag atggacaagc acaaccggcc
	721 acagctaatt actacattgt aacgtgttgt tacacttgtg acaccacggt tcgtttgtgt
	781 atcaacagta caacaaccga cgtacgaacc ctacagcagc tgcttatggg cacatgtacc
	841 attgtgtgcc ctagctgtgc acagcaataa acaccatctg caatggatga ccctgaaggt
	901 acaaacgggg taggggcggg ctgtactggc tggtttgagg tagaagcagt aatagaacga
	961 agaacaggag ataatatttc agatgatgag gacgaaacag cagacgatag tggtacagat
	1021 ttaatagagt ttatagatga ttcagtacaa agtactacac aggcagaagc agaggcagcc
	1081 cgagcgttgt ttaatgtaca ggaaggggtg gacgatataa atgctgtgtg tgcactaaaa
	1141 cgaaagtttg cagcatgctc agaaagtgct gtagaggact gtgtggaccg ggccgcaaat

1201 gtgtgtgtat cgtggaaata taaaaataaa gaatgcacac acagaaaacg aaaaattatt
1261 gagctagaag acagcggata tggcaatact gaagtggaaa ctgagcagat ggcacaccag
1321 gtagaaagcc aaaatggcga cgcagactta aatgactcgc agtctagtgg ggtgggggct
1381 agttcagatg taagcagtga aacggatgta gacagttgta atactgttcc attacaaaat
1441 attagtaata ttttacataa cagtaatact aaagcaacgc tattatataa attcaaagaa
1501 gcttatggag taagttttat ggaattagtt agaccattta aaagtgataa aacaagctgt
1561 acagattggt gtataacagg gtatggaata agtccctccg tagcagaaag tttaaaagta
1621 ctaattaaac agcacagtat atatacacac ctacaatgtt taacgtgtga cagaggaatt
1681 atattattat tgttaattag atttaaatgt agcaaaaata gattaactgt ggcaaaatta
1741 atgagtaatt tactatcaat tcctgaaaca tgtatgatta ttgagccacc aaaattacga
1801 agtcaagcat gtgccttata ttggtttaga acagcaatgt caaatataag tgatgtgcaa
1861 gggacaacac cagaatggat agatagatta acagtgttac agcatagctt taatgatgat
1921 atatttgatt taagtgaaat gatacaatgg gcatatgata atgacattac agatgatagt
1981 gacattgcat ataaatatgc acagttagca gatgttaata gtaatgcagc agcattttta
2041 agaagcaatg cacaagcaaa aatagtaaaa gactgtggcg ttatgtgcag acattataaa
2101 agagcagaaa agcgtggtat gacaatggga caatggatac aaagtaggtg tgaaaaaaca
2161 aatgatggag gtaattggag accaatagta caatttttaa gatatcaaaa tattgaattt
2221 acagcatttt tagttgcatt taaacagttt ttacaaggtg taccaaaaaa aagttgtatg
2281 ttactgtgtg gcccagcaaa tacagggaaa tcatattttg gaatgagttt aatacatttc
2341 ttaaaaggat gcattatttc atatgtaaat tccaaaagtc atttttggtt gcagccatta
2401 tcagatgcca aactaggtat gatagatgat gtaacagcca taagctggac atatatagat
2461 gattatatga gaaatgcatt agatggtaac gacatttcaa tagatgtaaa acatagggca
2521 ttagtacaat taaaatgtcc accattaata attacctcaa atacaaatgc aggcaaagat
2581 tcacgatggc catatttgca cagtagacta acagtatttg aatttaacaa tccatttcca
2641 tttgatgcaa atggtaatcc agtgtataaa ataaatgatg aaaattggaa atcctttttc
2701 tcaaggacgt ggtgcaaatt aggcttaata gaggaagagg acaaggaaaa cgatggagga
2761 aatatcagca cgtttaagtg cagtgcagga caaaatccta gacatatacg aagctgataa
2821 aaatgattta acatcacaaa ttgaacattg gaaactaata cgcatggagt gtgctataat
2881 gtatacagcc agacaaatgg gaatatcaca tttgtgccac caggtggtgc cgtccttggt
2941 agcatcaaag actaaagcgt ttcaagtaat tgaactgcaa atggcattag agacattaaa
3001 tgcatcacca tataaaacag atgaatggac attgcaacaa acaagcttag aagtgtggtt
3061 atcagagcca caaaaatgct ttaaaaaaaa aggcataaca gtaactgtac aatatgacaa
3121 tgataaagca aacacaatgg attatacaaa ttggagtgaa atatatatta ttgaggaaac
3181 aacatgtact ttggtagcag gagaagttga ctatgtgggg ttgtattata tacatggcaa
3241 tgaaaagacg tattttaaat attttaaaga ggatgcaaaa aagtactcta aaacacaatt
3301 atgggaggta catgtgggta gtcgggtaat tgtatgtcct acatctatac ctagtgatca
3361 aatatccact actgaaactg ctgacccaaa gaccaccgag gccaccaaca acgaaagtac
3421 acaggggaca aagcgacgac gactggattt accagactcc agagacaaca cccagtactc
3481 cacaaagtat acagactgcg ccgtggacag tagaccacga ggaggaggac tacacagtac
3541 aactaactgc acatacaaag ggcggaacgt gtgtagttct aaagttgcac ctatcttgca
3601 tttaaaaggt gacccaaata gtttaaaatg tttaagatat agattaaaac catttaaaga
3661 cttatactgt aatatgtcat ccacatggca ttggaccagt gatgacaaag gtgacaaagt
3721 aggaattgtt actgtaacat acacaacgga aacacaacga caactgtttt taaacactgt
3781 taaaatacca cccactgtgc aaataagtac tggtgttatg tcattgtaat tgtattgtac
3841 aattactgta tgtaaaccac aagccaatat gtgctgctaa gtgtatatac aatgatatta
3901 cctatttttg ttgtttgttt tatactgttt ttatgcttgt gcatttttct gcggccattg
3961 gtgctatcta tttctatata tgcttggctg ctggtgttgg tgttgctgct ttgggtgtct
4021 gtggggtcgg ctttacgaat ttttttctgt tacttaatat ttttatatat accaatgatg
4081 tgtattaatt ttcatgcaca atacttaacc caacaagact aactgtatac tgggtatgca
4141 catggtggta tggtattgta aatatttact gttgtgtgtg ttgtttttat tatttttata
4201 catttactaa taaatacttt tatattttta gcactgtctt attatgagac acaaacggtc
4261 tacaaggcgc aagcgtgcat ctgctacaca actttaccaa acatgcaagg cctcaggcac
4321 ctgcccacct gatgttatac ccaaagttga aggcactact atagcagatc aaatattacg
4381 atatggtagc ttaggggtgt tttttggagg tttaggcatt ggtacagggt cgggtacagg
4441 tggcaggact ggatatgtgc cccttggtag taccccaccg tctgaggcta tacctttaca
4501 gcccatacgt cccccagtta ccgttgatac tgtggggcct ttggattctt ctattgtatc
4561 tttaatagag gaatctagtt ttatagacgc cggtgcacca gccccatcaa ttcccactcc

	4621 atctggcttt gatattacca cctctgcaga tactacacct gcaatactta atgtttcctc
	4681 tattggagaa tcatctatac aaactgtttc tacacattta aatccctcct ttactgagcc
	4741 atccgtactc cgccctcctg cacctgcaga ggcctctgga catttaatat tttcctctcc
	4801 tactgttagc acacatagtt atgaaaacat accaatggat acctttgtta tttctactga
	4861 cagtggcaat gtcacgtcta gcacacccat tccagggtct cgccctgtgg cacgccttgg
	4921 tttatacagt cgcaacaccc aacaggttaa ggttgttgac cctgcttttt taacatctcc
	4981 tcatagactt gtaacatatg ataatccagc atttgaaggc tttaaccctg aggacacatt
	5041 gcagtttcaa catagtgaca tatcgcctgc tcctgatcct gattttctag atattgttgc
	5101 attgcacaga cctgcattga cctctcgcag gggtactgta cggtatagta gggttgggca
	5161 aaaggctaca cttcgtactc gcagtggaaa gcaaataggg gctaaagtac attactacca
	5221 agacttaagt cccatacagc ctgtccagga acaggtacaa cagcaacaac aatttgaatt
	5281 acaatcttta aatacttctg tttctcccta tagtattaat gatggacttt atgatattta
	5341 tgctgacgat gctgatacta tacatgattt tcagagtcct ctgcactcac atacctcctt
	5401 tgccaccaca cgtaccagta atgtgtccat accattaaat actggatttg acactcctct
	5461 tgtgtcattg gaacctggtc cagacattac atcttctgta acatctatgt ctagtccatt
	5521 tattcctata tctccactac ctccttttaa taccataatt gtggatggtg ctgattttat
	5581 gttgcaccct agctatttta ttttgcgtcg cagacgtaaa cgttttccat attttttgc
	5641 agatgtccgt gtggcggcct agtgaggcca ctgtgtacct gcctcctgtg cctgtgtcta
	5701 aggttgtaag cactgatgaa tatgtgtcac gcacaagcat ttattattat gctggcagtt
	5761 ccagactttt ggctgttggc aatccatatt tttccatcaa aagtcccaat aacaataaaa
	5821 aagtattagt tcccaaggta tcaggcttac agtatagggt ctttagggtg cgtttacctg
	5881 atcccaataa atttggtttt cctgatacat ctttttataa ccctgataca caacgtttgg
	5941 tctgggcatg tgtaggcctt gaaataggta ggggacagcc attgggtgtt ggcgtaagtg
	6001 gtcatcctta tttcaataaa tttgatgaca ctgaaaccag taacagatat cccgcacagc
	6061 cagggtctga taacagggaa tgcttatcta tggattataa acaaacacaa ttatgtttaa
	6121 ttggctgtaa acctcccact ggtgagcatt ggggtaaagg tgttgcctgt aacaataatg
	6181 cagctgctac tgattgtcct ccattggaac tttttaattc tattattgag gatggtgaca
	6241 tggtagatac agggtttgga tgcatggact ttggtacatt gcaggctaat aaaagtgatg
	6301 tgcctattga tatttgtaac agtacatgca aatatccaga ttatttaaaa atggccagtg
	6361 aaccttatgg ggatagtttg ttcttttttc ttagacgtga gcagatgttt gttaggcact
	6421 tttttaatag ggccggaaaa cttggcgagg ctgtcccgga tgacctttat attaaagggt
	6481 ccggtaatac tgcagttatc caaagtagtg catttttttcc aactcctagt ggctctatgg
	6541 ttacctcaga atcacaatta tttaataagc cttattggct acagcgtgca caaggtcata
	6601 acaatggcat ttgctggggc aatcagttat ttgttaccgt agttgatacc actcgtagca
	6661 ctaatatgac attatgcact gaagtaacta aggaaggtac atataaaaat gataatttta
	6721 aggaatatgt acgtcatgtt gaagaatatg acttacagtt tgtttttcag ctttgcaaaa
	6781 ttacactaac tgcagagata atgacatata tacatactat ggattccaat attttggagg
	6841 actggcaatt tggtttaaca cctcctccgt ctgccagttt acaggacaca tatagatttg
	6901 ttacctccca ggctattact tgccaaaaaa cagcaccccc taaagaaaag gaagatccat
	6961 taaataaata tactttttgg gaggttaact taaaggaaaa gttttctgca gatctagatc
	7021 agtttccttt gggacgaaag tttttattac aatcaggcct taaagcaaag cccagactaa
	7081 aacgttcggc ccctactacc cgtgcaccat ccaccaaacg caaaaaggtt aaaaaataat
	7141 tgttgtggta cttacactat tttattatac atgtttgttt gttttatgta tgtgttgtct
	7201 gtttgtttat gtttgtgtat atgttgtatg tgttatgtgt catgtttgtg tacatgttct
	7261 atgtgtttgt cagtttcctg tttctgtata tatgtaataa actattgtgt gtattgtaaa
	7321 ctatttgtat tgtttgggtg tatctatgag taaggtgctg tccctaaatt gccctaccct
	7381 gccctgccta ttatgcatac ctatgtaata gtatttgtat gatatgtatt ttatagtttt
	7441 taacagtact gcctccattt tactttacct ccattttgtg catgtaaccg atttcggttg
	7501 ctggcacaaa cgtgtttttt ttaaactaca atttaaacaa tacagttaat cctttccctt
	7561 cctgcactgc ttttgcctat acttgcatat gtgactcata tatacatgca gtgcagttgc
	7621 aaaatgttta attatactca tagtttaaac atgcttatag gcacatattt taacttactt
	7681 tcaatgctta agtgcagttt tggcttgcac aatggtttgt tatgccaaac tatgtcttgt
	7741 aaaagtgact cactaacatt tattgccagg tgtggactaa ccgttttggg tcacattgtt
	7801 catgtttcaa cattttatat aata
HPV 52	1 taaattataa tcctatacta gtaaaaaata gggtgtaacc gaaaacggtc agaccgaaac
	61 cggtgtatat atatagaaca cagtgtagct aacgcacggc catgtttgag gatccagcaa
	121 cacgaccccg gaccctgcac gaatgggtcg tgttgctgga agaatcggtg catgaaataa

181 ggctgcagtg tgtgcagtgc aaaaaagagc tacaacgaag agaggtatac aagtttctat
241 ttacagattt acgaatagta tatagagaca ataatccata tggcgtgtgt attatgtgcc
301 tacgcttttt atctaagata agtgaatata ggcattatca atattcactt tatgggaaaa
361 cattagaaga gagggtagga aaaccattaa gtgaaataac tattagatgt ataatttgtc
421 aaacgccatt atgtcctgaa gaaaaagaaa gacatgttaa tgcaaacaag cgatttcata
481 atattatggg tcgttggaca gggcgctgtt cagagtgttg gagaccccga cctgtgaccc
541 aagtgtaacg tcatgcgtgg agacaaagca actataaaag attatatatt agatctgcaa
601 cctgaaacaa ctgacctaca ctgctatgag caattaggtg acagctcaga tgaggaggat
661 acagatggtg tggaccggcc agatggacaa gcagaacaag ccacaagcaa ttactacatt
721 gtgacatatt gtcacagttg tgatagcaca ttacggctat gcattcatag cactgcgacg
781 gaccttcgta ctctacagca gatgctgttg ggcacattac aagttgtgtg ccccggctgt
841 gcacggctat aaacaaccct gcaatggagg accctgaagg tacagagggc gaaagggagg
901 gatgtacagg ctggtttgaa gtagaggcaa taatagaaga acaaacagga gataaccttt
961 ctgaggacga ggatgaaatt acatatgata gtggaacaga tataatagat tttatagatg
1021 attcaaatat aaataatgaa caggcagaac atgaggcagc ccgggcattg tttaatgcac
1081 aggaaggggga ggatgatgta catgctgtgt ctgcagtaaa acgaaagttt acaagcagtc
1141 cggaaagtgc tgggcaagat ggtgtagaaa aacatggtag tccgcgtgca aaacacattt
1201 gtgtaaatac agagtgtgtt ttaccaaaac gcaaaccatg tcaagtagaa gacagcggct
1261 atggcaatag tgaagtggaa gcgcagcaga tggcagacca ggtagacggg caaaatggcg
1321 actggcaaag taacagtagt caatcaagtg gggtgggggc tagtaattca gatgtaagtt
1381 gtactagtat agaggacaat gaggaaaata gtaatagaac gctaaaaagc atacaaaata
1441 ttatgtgcga aaatagcata aaaacaactg tattatttaa atttaaagaa acatatggtg
1501 ttagctttat ggaattagta agaccattta aaagtaatag aagtagttgc acagattggt
1561 gtattatagg aatgggagta acaccatcag ttgcagaagg attaaaagta ttaatacagc
1621 cctatagcat atatgcccat ttgcaatgtt taacatgtga cagaggcgtg cttatactgc
1681 tgctaattag gtttaaatgt gggaaaaaca gattaacagt gtccaaacta atgtcacagc
1741 tgttaaatat accagaaaca catatggtaa tagaaccacc aaaattacga agtgctacct
1801 gtgcattata ttggtataga acaggtttgt ctaatattag tcaggtatat ggtaccaccc
1861 cagaatggat agaacaccaa acagtattac agcatagctt tgacaatagc atatttgatt
1921 ttggagaaat ggtgcaatgg gcatatgatc atgatataac agatgatagt gacatagcat
1981 ataaatatgc acagttagca gatgtaaata gcaatgctgc agcattccta aaaagcaatt
2041 cgcaagcaaa aatagtaaag gactgtgcaa ccatgtgtag acattataaa cgggcagaaa
2101 gaaaacatat gaatattgga caatggatac agtatagatg tgataggata gatgatggtg
2161 gagattggag gcctatagta agatttttaa gatatcaaga catagaattt acagcctttt
2221 tagacgcatt taaaaaattt ttaaaaggta tacctaaaaa aaattgttta gtattatatg
2281 gacctgcaaa cacaggaaaa tcatattttg gaatgagttt aattaggttc ttaagtggat
2341 gtgtaatatc ctatgtaaac tcaaaaagcc atttttggct acaaccatta acagatgcaa
2401 aagtgggtat gatagatgat gtaacaccta tatgttggac atatatagat gattatatga
2461 gaaatgcact ggatggaaat gatatatcag tagatgtaaa gcatagagcc ttagtacaaa
2521 taaaatgccc accattaatt ttaacaacaa atacaaatgc aggaacagat cctaggtggc
2581 catatttaca tagtagattg gttgtgtttc atttcaaaaa cccatttcca tttgatgaaa
2641 atggcaatcc catatatgaa attaacaacg aaaattggaa atccttttc tcaaggacgt
2701 ggtgcaaatt agatttaata caggaagagg acaaggaaaa cgatggagtc gataccggca
2761 cgtttaaatg cagtgcagga aaaaatacta gatctatacg aagctgatag taatgaccta
2821 aacgcacaaa ttgaacattg gaaattgact cgaatggaat gtgttttgtt ttacaaagca
2881 aaggaactgg gaataactca tataggccac caggtggtgc caccaatggc agtgtctaag
2941 gcaaaggcct gccaagctat tgaactacaa ttggcattgg aggcattaaa caaaacacaa
3001 tatagcacag atggatggac attacaacaa acaagtctag aaatgtggcg tgcagaacca
3061 caaaaatact ttaaaaaaca tgggtataca ataacagtgc aatacgataa tgataaaaac
3121 aatactatgg attatacaaa ttggaaggaa atttatttac ttggtgagtg tgaatgtaca
3181 attgtagaag gacaagtaga ttattatggg ttatattatt ggtgtgatgg agaaaaaaca
3241 tattttgtaa aattcagtaa cgatgcaaag caatattgtg caacaggagt atgggaagta
3301 catgtgggtg gtcaggtaat tgtttgtcct gcatctgtat ctagtaacga agtatccact
3361 actgaaactg ctgtccacct atgcaccgaa acctccaaga cctccgcatt gtccgtgggt
3421 gccaaagaca cacacctaca accaccacag aaacgacgac gaccagacgt cacagactcc
3481 agaaacacca agtaccccaa caaccttttg cggggacaac aatccgtgga cagtactaca
3541 cggggactcg tcactgcaac tgagtgcaca aacaaaggac gggttgcaca tacaacttgt

3601 actgcaccta taatacacct aaaaggtgat cctaatagtt taaaatgttt aagatatagg
3661 gtaaaaccac ataacagttt gtatgttcaa atttcatcta cctggcattg gaccagtaat
3721 gaatgtacaa atactaaact aggtattgta acaataacat acagtgatga agcacaacgt
3781 gaacaatttt taaaaactgt taaaatacca aatactgtgc aagttataca aggtgtcatg
3841 tcattgtaat atttgtacat atgtatatat gtatatgtgt atggtaaaca cccaacacaa
3901 gccaatattg ctgctattgt gtatatagaa caatgttagg attatttgta ttttgtttta
3961 ttttgcttat ggtgttttgt gcagtgctta ggccgctctt gctatctata tcggtgtatg
4021 cgcaggtgtt ggtgctggtg cttttgctat gggtgtctat tgggtcacca tttaaagtgt
4081 ttttttgta cctactgttt ttatattttc caatgttttg tattcactgt catgcacagt
4141 atttggcaca actgcaataa ctattgtaca aaactgttgt gtgtactgct gtacatgtag
4201 attggctaca tgcatatatg caaaatatac ttttttactt ttgtagtttg tctaataaat
4261 acttttatat tttttaatag cttgtcgcaa tgagatacag acggtctaca cggcacaaac
4321 gtgcttctgc aacacagcta tatcaaacat gcaaagcctc tggcacctgc ccccccgatg
4381 ttattcctaa agtggaaggc acaactattg cagatcaact tttaaaatat ggcagcctag
4441 gggtgttttt tggaggtttg ggtataggta caggtgcagg ctctggtggt agggcgggct
4501 atgtgccttt gtccactcgt cctcccacta gtagtattac cacgtccact attcgtcccc
4561 ctgtaactgt agaacccatt ggtcccttag aaccatctat agtttctatg atagaagaaa
4621 caacatttat tgagtctggc gcacctgctc catctattcc atcagcaaca gggtttgatg
4681 ttacaacatc tgcaaataat actcctgcaa taattaatgt aacatctata ggtgaatcat
4741 ctgtacaatc agtttctaca catttaaatc ctacattcac tgaaccatct ataatacagc
4801 ccccggcacc tgcagaagca tctggtcatg tattgttttc tagtccaact attagtacac
4861 acacctatga agaaatccct atggatacat ttgttacctc tactgacagc agcagtgtaa
4921 caagtagtac acctattcca gggtctcgcc ctacgacacg ccttggttta tatagccgtg
4981 ccacacaaca ggttaaggta gtcgaccctg cttttatgtc atcaccacaa aaattagtaa
5041 catataacaa tcctgttttt gagggcgttg atacagatga aactataatt tttgatcgtt
5101 cacaactttt acctgccccg gatcctgatt ttttagacat tatagctttg cataggcctg
5161 cattaacctc tcgaagaggt actgttaggt ttagcaggct tggtaataag gccaccctac
5221 gtacacgtag tggaaaacaa attggggcac gggtacatta ttatcatgat attagtccta
5281 tccagcctgc tgaagttcag gaagacatag aattgcaacc tttattacca cagtctgtgt
5341 ccccttacac tattaatgat ggtttgtatg atgtgtatgc agagtctttg cagcaaccca
5401 cgtttcactt accttccaca ctttctaccc ataataatac tttcactgta cctattaata
5461 gtggtattga ctttgtatat caacccacta tgtccattga gtcaggtcct gacattccat
5521 taccttcgtt acccacacat actccttttg ttcctatagc ccctacagct ccatctacat
5581 ctattattgt tgatggtaca gattttattt tacatcctag ttatttttta ctacgtcgca
5641 ggcgtaaacg ttttccatat ttttttacag atgtccgtgt ggcggcctag tgaggccact
5701 gtgtacctgc ctcctgtacc tgtctctaag gttgtaagca ctgatgagta tgtgtctcgc
5761 acaagcatct attattatgc aggcagttct cgattactga cagtaggaca tccctatttt
5821 tctattaaaa acaccagtag tggtaatggt aaaaaagttt tagttcccaa ggtgtctggc
5881 ctgcaataca gggtatttag aattaaattg ccggacccta ataaatttgg ttttccagat
5941 acatcttttt ataacccaga aacccaaagg ttggtgtggg cctgtacagg cttggaaatc
6001 ggtaggggac agcctttagg tgtgggtatt agtgggcatc ctttattaaa caagtttgat
6061 gatactgaaa ccagtaacaa atatgctggt aaacctggta tagataatag ggaatgttta
6121 tctatggatt ataagcaaac tcagttatgc attttaggat gcaaacctcc tataggtgaa
6181 cattggggta agggaacccc ttgtaataat aattcaggaa atcctgggga ttgtcctccc
6241 ctacaactca ttaacagtgt aatacaggat ggggacatgg tagatacagg atttggttgc
6301 atggatttta ataccttgca agctagtaaa agtgatgtgc ccattgatat atgtagcagt
6361 gtatgtaagt atccagatta tttgcaaatg gctagcgagc catatggtga cagtttgttt
6421 tttttcttta gacgtgagca aatgtttgtt agacactttt ttaatagggc cggtacctta
6481 ggtgaccctg tgccaggtga tttatatata caagggtcta actctggcaa tactgccact
6541 gtacaaagca gtgctttttt tcctactcct agtggttcta tggtaacctc agaatcccaa
6601 ttatttaata aaccgtactg gttacaacgt gcgcagggcc acaataatgg catatgttgg
6661 ggcaatcagt tgtttgtcac agttgtggat accactcgta gcactaacat gactttatgt
6721 gctgaggtga aaaaggaaag cacatataaa aatgaaaatt ttaaggaata ccttcgtcat
6781 ggcgaggaat tcgatttaca atttattttt caattgtgca agattacatt aacagctgat
6841 gttatgacat atattcataa gatggatgcc actattttag aggactggca atttggcctt
6901 accccaccac cgtctgcatc tttggaggac acatacagat ttgtaacttc tactgctata
6961 acttgtcaaa aaaacacacc acctaaagga aaggaagatc ctttaaagga ctatatgttt

	7021 tgggaggtgg atttaaaaga aaagttttct gcagatttag atcagtttcc tttaggtaga
	7081 aagtttttgt tacaggcagg gctacaggct aggcccaaac taaaacgccc tgcatcatcg
	7141 gccccacgta cctccacaaa gaagaaaaag gttaaaaggt aaccattgtc tgttgcgtaa
	7201 ttgtctgtgt catgtatgtg ttgtgtatgt caaaaacagg ttaaaaggta accattgttt
	7261 gttatgtaat tgttttgtgt gtgtactgtg ttgtttgcat gttatgtatg tgtgcatgtt
	7321 tgttgtattt gtcagttcct gtatgtatgt tttgtgtatg tattaataaa gtactgtatt
	7381 tactaaacta tttatattag tcttatgtta tggttgcacc cacatgagta acaatacagt
	7441 tgctcctaat ctattgcatc tcctgcccta ccctgtgtcc cctgccctac cctgtgtcct
	7501 actttgttac actactaatt agccttatac tctccatttt gtaccatttt gtactatcca
	7561 ccattttaaa tcctaaccga attcggttgg tcttggcaca actttggttg tccttggcac
	7621 agtaacaact atttttatat aaatgtcagc aaactgctta atcctttggt ttcctgccgc
	7681 ccactggtct acacttgttg tcccgcctaa actgacttgc tgactcacac gtcctgcagt
	7741 gcagctaaac aatacattgc ctaacattgc atgttttaaa ctgcttttag gcacatattt
	7801 tatttaaact ttcaatgcac taattacagt gttggcttac acaagtacat cctacgccaa
	7861 atatgtcttg taaaacataa ttgaatactg ttactcacca ggtgtgcact acacgaccgg
	7921 ttacggttac cgtacccaca accacttttt tttataatta
HPV 31	1 taataataat aatcttgtta taaaaaagta gggagtaacc gaaattggtg aaccgaaaac
	61 ggttggtata taaagcacat agtattttgt gcaaacctac agacgccatg ttcaaaaatc
	121 ctgcagaaag acctcggaaa ttgcatgaac taagctcggc attggaaata ccctacgatg
	181 aactaagatt gaattgtgtc tactgcaaag gtcagttaac agaaacagag gtattagatt
	241 ttgcatttac agatttaaca atagtatata gggacgacac accatacgga gtgtgtacaa
	301 aatgtttaag attttattct aaagtaagtg aatttagatg gtatagatat agtgtgtatg
	361 gaacaacatt agaaaaattg acaaacaaag gtatatgtga tttattaatt aggtgtataa
	421 cgtgtcagag accgttgtgt ccagaagaaa aacaaagaca tttggataaa aagaaacgat
	481 tccacaacat aggaggaagg tggacaggac gttgcatagt atgttggaga agacctcgta
	541 ctgaaaccca agtgtaaaca tgcgtggaga aacacctaca ttgcaagact atgtgttaga
	601 tttgcaacct gaggcaactg acctccactg ttatgagcaa ttacccgaca gctcagatga
	661 ggaggatgtt atagacagtc cagctggaca agcaaaaccg gacacatcca attacaatat
	721 cgttaccttt tgttgtcagt gtgagtctac acttcgtttg tgtgtacaga gcacacaagt
	781 agatattcgc atattgcaag agctgttaat gggctcattt ggaatcgtgt gccccaactg
	841 ttctactaga ctgtaactac aatggctgat ccagcaggta cagatgggga ggggacggga
	901 tgcaatggtt ggttttatgt agaagcagta attgacagac agacagggga caacatttca
	961 gacgacgaaa atgaagacag tagtgatact ggggaggata tggttgactt tattgacaat
	1021 tgtaatgtat acaacaatca ggcagaagca gagacagcac aggcattgtt tcatgcacag
	1081 gaagcggagg aacatgcaga ggctgtgcag gttctaaaac gaaagtatgt aggtagtcct’
	1141 ttaagtgata ttagtagttg tgtggattat aatattagtc cacggttaaa agctatatgc
	1201 atagaaaata acagtaaaac agcaaaacga agactttttg aacttccaga cagcgggtat
	1261 ggcaatactg aagtggaaac gcagcagatg gtacaggtag aggagcaaca aacaacatta
	1321 agttgtaatg gtagtgacgg gacacatagt gaacgagaca atgaaactcc aacacgtaat
	1381 atattgcaag tgttaaaaac tagcaatggt aaagctgcta tgttaggtaa atttaaagaa
	1441 ttatatggtg taagttttat ggaactaatt aggccatttc aaagcaataa aagcacatgt
	1501 actgattggt gtgtagctgc gtttggagtt acaggtacag ttgcagaagg atttaaaacc
	1561 ctattgcaac catattgttt gtattgccat ttacaaagtt tagcatgttc ctggggcatg
	1621 gttatgttaa tgcttgtaag atttaaatgt gcaaaaaata gaataacaat tgaaaaatta
	1681 ttagaaaagt tattgtgtat atctacaaat tgtatgttaa ttcagccacc caaattacgt
	1741 agtacagctg cagcattata ttggtacaga acaggaatgt caaacattag tgatgtatat
	1801 ggtgaaacac cagaatggat agaaagacaa acagtattac agcatagttt taatgacaca
	1861 acatttgatt tgtcccaaat ggtacaatgg gcatatgaca atgatgttat ggatgatagt
	1921 gaaattgcct atagatatgc acaattagct gacagtgata gtaatgcatg tgcattttta
	1981 aaaagtaatt cgcaggcaaa aatagttaaa gattgtggaa caatgtgtag acattataaa
	2041 cgagcagaaa aacgacaaat gtctatggga cagtggatta aaagtagatg tgacaaagtt
	2101 agtgacgaag gtgactggag ggacatagta aagtttttaa gatatcaaca aatagaattt
	2161 gtgtcatttt tatctgcatt aaagctgttt ttaaaaggag tgccaaagaa aaactgtatt
	2221 ttaatacatg gtgcacctaa tacaggtaaa tcatattttg gaatgagcct tatgagcttc
	2281 ttacaaggat gtataatatc atatgcaaat tcaaaaagtc atttttggtt acaaccactg
	2341 gctgatgcta aaataggcat gttagatgat gctacaacgc catgttggca ttatatagac
	2401 aattacctac gaaatgcact agatggcaac cctgtatcta tagatgtaaa gcataaagct

2461 ttaatgcagt taaaatgtcc tcctttattg attacatcta atataaatgc aggtaaggat
2521 gacagatggc catacctaca tagcagactg gtggttttta catttccaaa tccatttcca
2581 tttgacaaaa acggaaatcc agtatatgaa ttaagtgata aaaactggaa atcctttttc
2641 tcaaggacgt ggtgcagatt aaatttgcac gaggaagagg acaaagaaaa cgatggagac
2701 tctttctcaa cgtttaaatg tgtgtcagga caaaatatta gaacattatg aaaatgatag
2761 taaacgactt tgtgatcata tagactattg gaaacatatt cgacttgaat gtgtattaat
2821 gtataaagca agagaaatgg gaatacacag tattaaccac caggtggtgc cagcgttgtc
2881 agtatcaaag gccaaagcct tacaagctat tgaactacaa atgatgttgg aaacattaaa
2941 taacactgaa tacaaaaatg aggactggac aatgcagcaa acaagtcttg aactgtattt
3001 aactgcacct acagggtgtt taaaaaaaca tggatatact gtacaggtgc aatttgatgg
3061 tgatgtacac aacaccatgc attatactaa ctggaaattt atatacctat gtatagatgg
3121 ccaatgtact gttgtggaag ggcaagttaa ttgtaagggc atttattatg tacatgaagg
3181 acatataaca tattttgtaa attttacaga agaggcaaaa aaatatggga ctggtaaaaa
3241 atgggaagtg catgcgggtg gtcaggtaat tgtttttcct gaatctgtat ttagcagtga
3301 cgaaatatcc tttgctggga ttgttacaaa gctaccaaca gccaacaaca ccaccacatc
3361 gaattccaaa acctgcgcct tgggcaccag tgaaggtgtg cggcgggcga cgacgtctac
3421 taagcgacca agaacagagc cagagcacag aaacacccac caccccaaca agttgttgcg
3481 aggcgactcc gtggacagtg tcaactgtgg ggttatcagt gcagctgcat gcacaaacca
3541 aacaagggct gtcagttgtc ctgcaactac acctataata cacttaaaag gtgatgcaaa
3601 tatattaaaa tgtttaagat ataggctgtc aaaatataaa caattgtatg aacaagtgtc
3661 atctacatgg cattggacat gtacagatgg aaaacataaa aatgctattg taaccttaac
3721 atatataagt acatcacaaa gagacgattt tttaaatact gtaaaaatac ctaacacagt
3781 atcagtgtca acaggatata tgactattta gcctaatgat tgaactaaat atttctacag
3841 taagcattgt gctatgcttt ttgctttgct tttgtgtgct actatttgtg tgtcttgtca
3901 tacgtccact tgtgctgtct gtgtcggtat atgcaacact actattatta attgtggttt
3961 tatgggttat tgcaacctca ccattacgtt gtttttgtat atatgttgtg tttatatata
4021 ttccattatt tgtaattcat acacatgcat ccattttaag tcaacagtaa ctttttttact
4081 tgtatatact gttgtttgta ttggtattgg tataataaac ttttttactt tttttttatt
4141 attaccatgc ggtccaaacg ctctacaaaa cgcactaaac gtgcgtctgc tacacaatta
4201 tatcaaacat gtaaagcagc aggtacttgt ccatcagacg ttatacctaa aatagaacat
4261 actaccattg cagaccaaat attaaggtat ggtagtatgg gtgtgttttt tggtgggttg
4321 ggtattgggt ccggatctgg tactgggggt cgcactggat atgtccctct tagtacacgt
4381 ccttctacag tatctgaggc aagtatacct attagaccac cagttagcat tgaccctgta
4441 ggtcccttgg acccctctat agtaagtctt gttgaagaat ctggaattat tgatgttggt
4501 gcccctgctc ctataccaca ccctcctaca acatctgggt ttgacattgc tacaactgca
4561 gacacaacac ctgcaatttt agatgtaaca agtgttagca cacatgagaa tcctactttc
4621 actgatccat ctgtattgca acctcctaca cctgcagaaa catcaggtca tttactactt
4681 tcatcatcat ctattagcac acataattat gaggaaatac ctatggatac atttattgtt
4741 tctactaata atgaaaacat aacaagtagc acacccattc caggggtgcg ccgtcctgca
4801 cgtttagggt tatatagtaa ggctacacaa caagtaaaag ttattgatcc aacgtttctt
4861 agtgctccaa aacaactaat tacatatgaa aaccctgcct atgaaactgt aaatgctgaa
4921 gaatctttat actttttccaa tacatcgcat aatatggccc ctgatcccga ctttttagat
4981 attatagcat tacataggcc tgcccttacc tcacgtagga acactgttag atatagtaga
5041 ctaggtaata aacaaacttt gcgcactcgt agtggtgcta ctattggtgc aagggtacat
5101 tattattatg atattagtag tattaatcct gcaggtgaaa gtattgaaat gcaaccttta
5161 ggggcgtctg caactactac ttctacttta aatgatggct tatatgacat ttatgcagac
5221 actgatttta ctgtggatac acctgccaca cataatgttt ccccttctac tgctttacag
5281 tccacatctg ctgtgtctgc ctatgtacct acaaatacca ctgtgccact aagtacaggt
5341 tttgacattc ccatattttc tgggcctgat gtacctatag agcatgcacc tacacaggtt
5401 tttccatttc ctttggcccc tacaacgcca caagtgtcta ttttgttga tgggggtgat
5461 ttttatttgc accctagtta ttatatgtta aaacgtcgac gtaaacgtgt atcatatttt
5521 tttacagatg tctctgtggc ggcctagcga ggctacagtc tacttaccac ctgtcccagt
5581 gtctaaagtt gtaagcacgg atgaatatgt aacacgaacc aacatatatt atcacgcagg
5641 cagtgctagg ctgcttacag taggccatcc atattattcc atacctaaat ctgacaatcc
5701 taaaaaaata gttgtaccaa aggtgtcagg attacaatat agggtattta gggttcgttt
5761 accagatcca aacaaatttg gatttcctga tacatctttt tataatcctg aaactcaacg
5821 cttagtttgg gcctgtgttg gtttagaggt aggtcgcggg cagccattag gtgtaggtat

	5881 tagtggtcat ccattgctaa ataaatttga tgacactgaa aactctaata gatatgccgg
	5941 tggtcctggc actgataata gggaatgtat atcaatggat tataaacaaa cacagctgtg
	6001 tttacttggt tgcaaaccac ctattggaga gcattggggt aaaggtagtc cttgtagtaa
	6061 taatgctatt acccctggtg attgtcctcc attagaatta aaaaattcag ttatacaaga
	6121 tggggatatg gttgatacag gctttggagc tatggatttt actgctttac aagacactaa
	6181 aagtaatgtt cctttggaca tttgtaattc tatttgtaaa tatccagatt atcttaaaat
	6241 ggttgctgag ccatatggcg atacattatt tttttattta cgtagggaac aaatgtttgt
	6301 aagacatttt tttaatagat caggcgcggt tggtgaatcg gttcctaatg acttatatat
	6361 taaaggctcc ggttcaacag ctactttagc taacagtaca tactttccta cacctagcgg
	6421 ctccatggtt acttcagatg cacaaatttt taataaacca tattggatgc aacgtgctca
	6481 gggacacaat aatggtattt gttggggcaa tcagttattt gttactgtgg tagataccac
	6541 acgtagtacc aatatgtctg tgtgtgctgc aattgcaaac agtgatacta catttaaaag
	6601 tagtaatttt aaagagtatt taagacatgg tgaggaattt gatttacaat ttatatttca
	6661 gttatgcaaa ataacattat ctgcagacat aatgacatat attcacagta tgaatcctgc
	6721 tattttggaa gattggaatt ttggattgac cacacctccc tcaggttctt tagaggatac
	6781 ctataggttt gtaacctcac aggccattac atgtcaaaaa actgcccccc aaaagcccaa
	6841 ggaagatcca tttaaagatt atgtattttg ggaggttaat ttaaaagaaa agttttctgc
	6901 agatttagat cagtttccac tgggtcgcaa atttttatta caggcaggat atagggcacg
	6961 tcctaaattt aaagcaggta aacgtagtgc accctcagca tctaccacta caccagcaaa
	7021 acgtaaaaaa actaaaaagt aatggatgtg tatgtaatac atgtgtctgt atgtgtatgt
	7081 gcttgtgctg tattgtatat gtgtgtgttt gtgtgttata tatggtgtat gtatgtttat
	7141 gtatgcgtgt gtacctgtat atatgtatag tatgttatgt gtgtatgtat gctatgtatg
	7201 ttaataaata tgtgtatacc tgtgtgtgtt gtgtatgttg tccttatata caccctatta
	7261 gtaacatact attactattt cataaactat tgttcctact tgtccctact cctcccaatg
	7321 gtcatgtact tattcctccc tataatttca gtgtcacgca atagtaaaag ttgtacaccc
	7381 ggtccgtttt ttgcaactaa agctactcca ttttaatttt atacagccat tttaaatcct
	7441 taaccgtttt cggttgcatt gtttaaacat gctagtacaa atatgctgat acagtagtcc
	7501 tgcggttttt ggtttcctga atactagttt ttgccaacat cctggcttgt agtttcctgc
	7561 ctaacacacc ttgccaacat ataatccagt ccaactttgc aattataata tgaatcatgt
	7621 ttgtgtaaat acaactgtag ttcaactatg tgtcatgcac atatattata ttgtcctaca
	7681 caccttaaac tgcttttagg cacatatttt gtagcttatc tatatccttg attgcagtgc
	7741 tggcttttgc acatgtttaa actgccaagg ttgtgtcatg cattataaat aagttgtatg
	7801 ttactcatat aattaattgc atataggtat tacaccgttt tcggttacag ttttacaagc
	7861 aattgttctt tttatact
HPV45	1 aatactttta acaattatac tacataaaaa agggtgtaac cgaaaacggt tgcaaccaaa
	61 aacggtgcat ataaaagctt tgtggaaaag tgcattacag gatggcgcgc tttgacgatc
	121 caaagcaacg accctacaag ctaccagatt tgtgcacaga attgaataca tcactacaag
	181 acgtatctat tgcctgtgta tattgcaaag caacattgga acgcacagag gtatatcaat
	241 ttgcttttaa agatttatgt atagtgtata gagactgtat agcatatgct gcatgccata
	301 aatgtataga cttttattcc agaattagag aattaagata ttattcaaac tctgtatatg
	361 gagagacact ggaaaaaata actaatacag agttgtataa tttgttaata aggtgcctgc
	421 ggtgccagaa accattgaac ccagcagaaa aacgtagaca ccttaaggac aaacgaagat
	481 ttcacagcat agctggacag taccgagggc agtgtaatac atgttgtgac caggcacggc
	541 aagaaagact tcgcagacgt agggaaacac aagtatagca ataagtatgc atggaccccg
	601 ggaaacactg caagaaattg tattgcattt ggaacctcag aatgaattag atcctgttga
	661 cctgttgtgt tacgagcaat taagcgagtc agaggaggaa aacgatgaag cagatggagt
	721 tagtcatgca caactaccag cccgacgagc cgaaccacag cgtcacaaaa ttttgtgtgt
	781 atgttgtaag tgtgacggca gaattgagct tacagtagag agctcggcag aggaccttag
	841 aacactacag cagctgtttt tgagcacctt gtcctttgtg tgtccgtggt gtgcaactaa
	901 ccaataatct acaatggcgg atccagaagg taccgacggg gagggaacgg ggtgtaatgg
	961 ctggttcttt gtagaaacaa ttgtagagaa aaaaacaggg gatgtaatat cagatgatga
	1021 ggatgaaact gcaacagata cagggtcgga tatggtagat tttattgaca cacaattatc
	1081 catttgtgaa caggcagagc aagagacagc acaggcattg ttccatgcgc aggaagttca
	1141 gaatgatgca caggtgttgc atcttttaaa acgaaagttt gcaggaggca gcaaggaaaa
	1201 cagtccatta ggggagcagc taagtgtgga tacggatcta agtccacggt tacaagaaat
	1261 ttcattaaat agtgggcaca aaaaagcaaa acgacggttg tttacaatat cagatagtgg
	1321 ctatggctgt tctgaagtgg aagctgcaga gactcaggta actgtaaaca ctaatgcgga

1381 aaatggcggc agtgtacata gtacacaaag tagtggtggg gatagtagtg acaatgcaga
1441 aaatgtagat ccgcattgca gtattacaga actaaaggag ctattacaag caagtaacaa
1501 aaaggctgca atgctggcag tatttaaaga catatatggg ctgtcattta cggatttggt
1561 tagaaatttt aaaagtgata aaacaacatg tacagattgg gtaatggcta tatttggagt
1621 taatccaacg gtagcagaag gctttaaaac attaattaaa ccagcaacgt tatacgccca
1681 tatccaatgt ttagattgta aatggggagt attaatatta gctttattaa gatataaatg
1741 tggcaaaaat agactaactg ttgcaaaagg cttaagcaca ttgttgcacg tacctgaaac
1801 atgtatgtta attgaaccac caaaattgcg aagtagtgtt gcagcattat actggtatag
1861 aacaggtata tccaatatta gtgaagtaag tggagacaca cctgagtgga tacaaagact
1921 gacaattatt caacatggta ttgacgatag taattttgat ttgtcagaca tggtgcaatg
1981 ggcatttgat aatgacctta cagatgaaag tgatatggca tttcaatatg cccaattagc
2041 agactgcaac agtaatgcag ctgcattttt aaaaagtaac tgccaagcca aatatttaaa
2101 agattgtgct gtaatgtgta gacattataa aagagcacaa aaacgccaaa tgaatatgtc
2161 tcaatggatt aaatatagat gttccaaaat agatgaaggt ggggattgga gacccatagt
2221 acaattccta agatatcagg gagtagaatt tattagcttt ttaagggcac taaaggaatt
2281 tcttaaagga acaccaaaaa aaaattgtat actgttatat ggacctgcaa atacaggaaa
2341 atcgtatttt ggaatgagtt ttatacattt cctacaaggt gcaataatat catttgtaaa
2401 ttcaaacagc catttttggt tagaaccgtt agcagatact aaggtagcca tgttggatga
2461 tgccacacac acgtgttgga catattttga taattatatg agaaatgcat tagatggtaa
2521 tcctataagt atagacagaa agcataaacc attattacag ctaaaatgtc ctccaatcct
2581 attaacatcc aatattgatc cagcaaaaga taataaatgg ccatatttag aaagtagggt
2641 gacggtattt acatttccac atgcatttcc atttgataaa aatggtaatc cagtatatga
2701 aataaatgat aaaaattgga aatgtttttt tgaaaggaca tggtccagat tagatttgca
2761 cgaggacgat gaagatgcag acaccgaagg aatccctttc ggaacgttta agtgcgttac
2821 aggacaaaat actagaccac tatgaaaatg acagtaaaga cataaacagc caaataagtt
2881 attggcaact tatacgttgg gaaaatgcaa tactatttac agcaagggaa catggtatta
2941 ccaaactaaa ccaccaggtg gtgcctccta ttaacatttc aaaaagcaaa gcacataaag
3001 ctattgaact gcaaatggcc ttaaagggcc ttgcacaaag caagtataac aatgaggaat
3061 ggacactgca agatacatgc gaggaactat ggaatacaga accgtcgcag tgttttaaaa
3121 aaggcggtaa aaccgtgcac gtatactttg atggcaacaa ggacaactgt atgaactatg
3181 tagtatggga cagtatatat tatataactg agacagggat atgggaaaaa acagcagcat
3241 gtgttagcta ttggggtgta tattatataa aagatggaga taccacatat tatgtacaat
3301 ttaaaagcga atgtgagaaa tatggaaata gtaatacgtg ggaagtacaa tatgggggca
3361 atgtaattga ttgtaatgac tctatgtgca gtaccagtga cgacacggta tccgctactc
3421 agattgttag acagctacaa cacgcctcca cgtcgacccc caaaaccgca tccgtgggca
3481 ccccaaaacc ccacatccag acgccggcta ctaagcgacc tagacagtgt ggactcacag
3541 agcagcacca cggacttgtc aacacccacg tgcacaaccc gctcctgtgt tcaagtacaa
3601 gtaacaacaa aagaaggaaa gtgtgtagtg gtaacactac gcctataata cacttaaaag
3661 gtgacaaaaa cagtttgaaa tgtttaagat ataggctacg caaatatgca gaccattact
3721 cagaaatatc ctccacctgg cattggacag gttgtaataa aaacactggt atattaactg
3781 taacatataa tagtgaggta caaagaaata ccttttgga tgtagttact attcctaaca
3841 gtgtacaaat ctcggtggga tacatgacta tatgaatctg tatattgtat acagtatgta
3901 acattactat gctatcttta gtgtttttat tgtgcttttc tgtgtgcctt tatgtgtgct
3961 gcaatgtccc gcttgtgcag tctgtctatg tgtgtgcttt cgcttggttg ttggtgtttc
4021 tttttatagt tgttattaca tccccattaa cagcatttgc tgtatacatt tgttgctatt
4081 tactacctat gtttgtatta catatgcatg ctttacacac catacaataa ttactataat
4141 gtacagtaca gtgtaacata cctgtgatgt gcatgttgtt gtatttttgt atttttgtat
4201 ttttgtattt ttgtattttt gtattttata tgtttaataa accatggtat cccaccgtgc
4261 agcacgtcgc aagcgggcct ctgcaactga cttatataga acatgtaagc aatccggtac
4321 gtgcccccct gatgttatta acaaagtgga aggcacaacc ttagctgata aaattttaca
4381 gtggtctagc cttgggatat ttttgggtgg ccttggcatt ggtaccggca gtggttctgg
4441 aggccgtacg ggctatgtac ccttaggggg caggtctaat actgttgtgg atgttggccc
4501 cactaggcca cctgtggtta ttgaacctgt agggcctact gatccatcta ttgttacgtt
4561 ggtagaggat tccagtgttg ttgcctctgg tgctccggtt cccacattta ccggaacctc
4621 tgggtttgaa attacgtctt ctggtactac cacaccagct gtgttggaca tcacacctac
4681 cgtggactct gtttctattt cgtcaactag ttttacaaat cctgcatttt ctgatccctc
4741 tattattgag gtgccccaaa caggggaggt atcaggtaat atatttgttg gtacaccaac

4801 atcgggcagc catggatatg aggaaatacc tttacaaaca tttgcatctt ctgggtcagg
4861 tacggaaccc attagtagta cccccctccc tactgtgcgg cgggtagcgg gtccccgcct
4921 gtatagtagg gctaatcaac aggtccgtgt gtccacctca cagtttttaa cacatccctc
4981 atcgttggtt acatttgata atccagctta tgagcccctg gacaccacac tatcctttga
5041 gcctaccagt aatgttcctg attccgattt tatggatatt attcgtttgc ataggccagc
5101 attatcctct agacgtggca ctgttagatt tagtagattg ggtcaaaggg caaccatgtt
5161 tacacgtagt ggtaaacaaa tagggggtag ggtacatttt taccatgata taagccccat
5221 tgctgctaca gaggaaattg aattgcagcc tttaattagt gctacagatg atagtgacct
5281 gtttgatgta tatgcagact tcccacctcc tgcgtccact acacctagca ctataaacaa
5341 atcatttaca tatccaaggt attccttgac catgccttct actgctgcat cctcttacag
5401 taatgttaca gtaccattaa catctgcatg ggatgtacct atatatactg gcccggacat
5461 tatattgcca tcccatactc ctatgtggcc tagtacatct cctaccaatg cttccaccac
5521 cacctatata ggtattcatg gcacacaata ttatttatgg ccatggtatt attattttcc
5581 taaaaaacgt aaacgtattc cctatttttt tgcagatggc tttgtggcgg cctagtgaca
5641 gtacggtata tcttccacca ccttctgtgg ccagagttgt caacactgat gattatgtgt
5701 ctcgcacaag catattttat catgcaggca gttcccgatt attaactgta ggcaatccat
5761 attttagggt tgtacctaat ggtgcaggta ataaacaggc tgttcctaag gtatccgcat
5821 atcagtatag ggtgtttaga gtaactttac ccgatcctaa taaatttgga ttacctgatt
5881 ctactatata taatcctgaa acacaacgtt tggtttgggc atgtgtaggt atggaaattg
5941 gtcgtgggca gcctttaggt attggcctaa gtggccatcc attttataat aaattggatg
6001 atacagaaag tgctcatgca gctacagctg ttattacgca ggatgttagg gataatgtgt
6061 cagttgatta taagcaaaca cagctgtgta ttttaggttg tgtacctgct attggtgagc
6121 actgggccaa gggcacactt tgtaaacctg cacaattgca acctggtgac tgtcctcctt
6181 tggaacttaa aaacaccatt attgaggatg gtgatatggt ggatacaggt tatggggcaa
6241 tggattttag tacattgcag gatacaaagt gcgaggttcc attagacatt tgtcaatcca
6301 tctgtaaata tccagattat ttgcaaatgt ctgctgatcc ctatggggat tctatgtttt
6361 tttgcctacg ccgtgaacaa ctgtttgcaa gacatttttg gaatagggca ggtgttatgg
6421 gtgacacagt acctacggac ctatatatta aaggcactag cgctaatatg cgtgaaaccc
6481 ctggcagttg tgtgtattcc ccttctccca gtggctctat tattacttct gattctcaat
6541 tatttaataa gccatattgg ttacataagg cccagggcca taacaatggt atttgttggc
6601 ataatcagtt gtttgttact gtagtggaca ctacccgcag tactaattta acattatgtg
6661 cctctacaca aaattctgtg ccaagtacat atgaccctac taagtttaag cagtatagta
6721 gacatgtgga ggaatatgat ttacagttta tttttcagtt gtgcactatt actttaactg
6781 cagaggttat gtcatatatc catagtatga atagtagtat attagaaaat tggaattttg
6841 gtgtccctcc accacctact acaagtttgg tggatacata tcgttttgtg caatcagttg
6901 ctgttacctg tcaaaaggat actacacctc cagaaaagca ggatccatat gataaattaa
6961 agttttggac tgttgaccta aaggaaaaat tttcctccga tttggatcaa tatccccttg
7021 gtcgaaagtt tttagttcag gctgggttac gtcgtaggcc taccatagga cctcgtaagc
7081 gtcctgctgc ttccacgtct actgcatcta ctgcatctag gcctgccaaa cgtgtacgta
7141 tacgtagtaa gaaataatat gttagcacat atatgtatgt ttgtatgtat ggttttgtat
7201 gttgtatgta tgtatgtatt tgtgtgatat attactgtat tttgtttgtt tgcgtgcgtg
7261 tatgtatgaa tgtgccttgt ggcatgtatg gtgttactgt acataattgt ggtattaaat
7321 aaagtatgct aatagtgttg tgtagggttg cacccttgtg agtaacaata ctatttgtgt
7381 gtatgtgtat tgctttgtac cctatattct ttcctgtatt tcaagttata aacttgcata
7441 ctacacagca tccattttac ttataatcct ccattttgct gtgcaaccga tttcggttgc
7501 ctgtggctta tatgtgacct tttaaacata atacctaaac tggcacattt acaaccccta
7561 catagtttaa cctactggcg cgccttcttg gcgtacatgt ggcacacctg gtattagtca
7621 ttttcctgtc caggtgtact aaaacaatgg cttgcacaac tgtatccaca ccctatgtaa
7681 taaaactgct tttaggcaca tattttagtc tgtttttacc tgtgctaatt gtataattgg
7741 cgtgtagaac cactttctta tccaacaatc tgtctacttg ttacataaac tataaactga
7801 ctcacttata catacatagt ttatgcaacc gaaaaaggtt gggccctata acacatacct
7861 tttctt

Supplementary Table S5

The qRT-PCR primers used in the study.

KRT14	Forward	GGCCTGCTGAGATCAAAGAC
	Reverse	GGCTCTCAATCTGCATCTCC
S100A8	Forward	TATCATCGACGTCTACCACAAG
	Reverse	TCTGCACCCTTTTTCCTGATAT
CLDN3	Forward	ATCATCACGTCGCAGAACATC
	Reverse	TACACCTTGCACTGCATCTG
TP63	Forward	TTCATGTGTAACAGCAGTTGTG
	Reverse	GCTGCTTTCTGATGCTATCTTC
GAPDH	Forward	ATGGGGAAGGTGAAGGTCG
	Reverse	GGGGTCATTGATGGCAACAATA
ROCK1	Forward	GCTGAGCAATATTTCTCGACAC
	Reverse	TCCAACTGAGTAGCAAGAGTTT
RHOA	Forward	AGGAAGATTATGATCGCCTGAG
	Reverse	CTAAACTATCAGGGCTGTCGAT
MMP7	Forward	AAATGCCAACAGTTTAGAAGCC
	Reverse	ATTATTTCTATGACGCGGGAGT
CDC42	Forward	GCTTGTTGGGACTCAAATTGAT
	Reverse	CCTTTCTGTGTAAGTGCAGAAC

Table S6

Antibodies used for flow cytometry analysis.

**Source**	**Identifier**	**Fluorescence**	**Antibody**
BD Biosciences	563024	BV510	Mouse anti-CD3
Biolegend	100722	PC5.5	Mouse anti-CD8
BD Biosciences	563757	BV650	Mouse anti-Ki67
BD Biosciences	563376	BV421	Mouse anti-IFNG
BD Biosciences	153406	PE	Mouse anti-PRF1

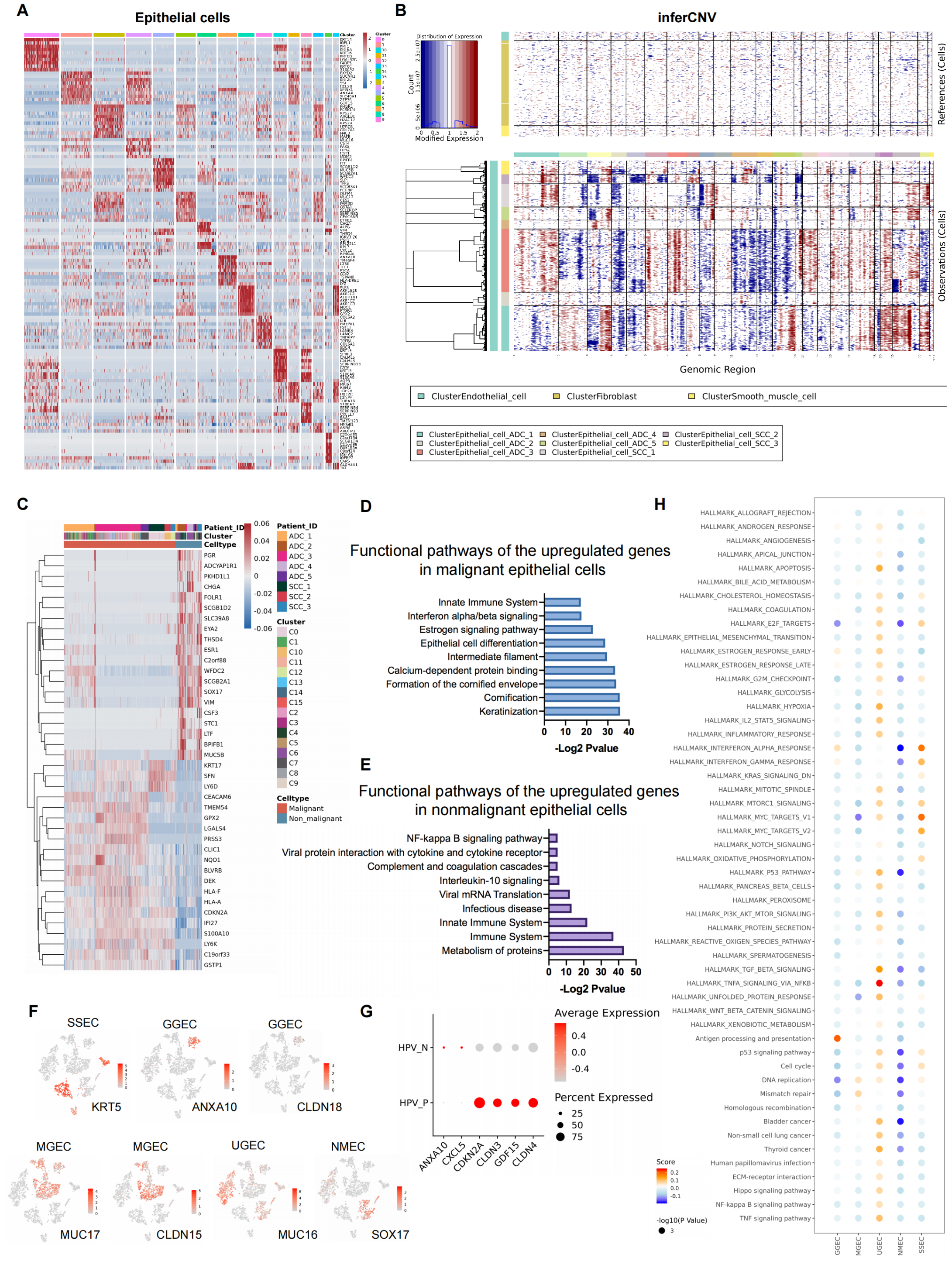

Figure S1. Features, classification and functions of the epithelial cells in CC TME

(A)Heatmap of marker gene expression of the 16 subclusters of epithelial cells.

(B) Inferred copy number variation (CNV) levels of the epithelial cells of 8 CC samples taking endothelial cells, fibroblasts and smooth muscle cells as reference.

(C) Heatmap indicating the DEGs expression between malignant and nonmalignant epithelial cells.

(D) Bar chart showing the enrichment of specific pathways, based on the GO (gene ontology)

pathways of upregulated genes in malignant epithelial cells presented with statistical significance [(-Log2(P value)].

(E) Bar chart showing the enrichment of specific pathways, based on the GO (gene ontology) pathways of upregulated genes in nonmalignant epithelial cells presented with statistical significance [(-Log2(P value)].

(F) Expression levels of representative markers of the 5 major types of epithelial cells are plotted onto the t-SNE map: KRT5 for SSECs (subclusters 0, 10, 12); ANXA10 and CLDN18 for GGECs (subcluster 7); MUC17 and CLDN15 for MGECs (subclusters 2, 5, 8, 9, 13, 15); MUC16 for UGECs (subclusters 1, 3, 11); and SOX17 for NMECs (subclusters 4, 6, 14). Color key from gray to red indicated relative expression levels from low to high.

(G) Dot plot indicating the average expression levels and cell expressing proportion of DEGs (differentially expressed genes) between HPV-positive and HPV-negative cells. The colors represented the average expression levels, and dot sizes represented the percentage expression of selected genes.

(H) Bubble plot of the enriched pathways of each major epithelial type based on HALLMARK gene set through QUSAGE analysis presented with statistical significance [(-Log10(P value)] and enrichment score (color key from blue to red).

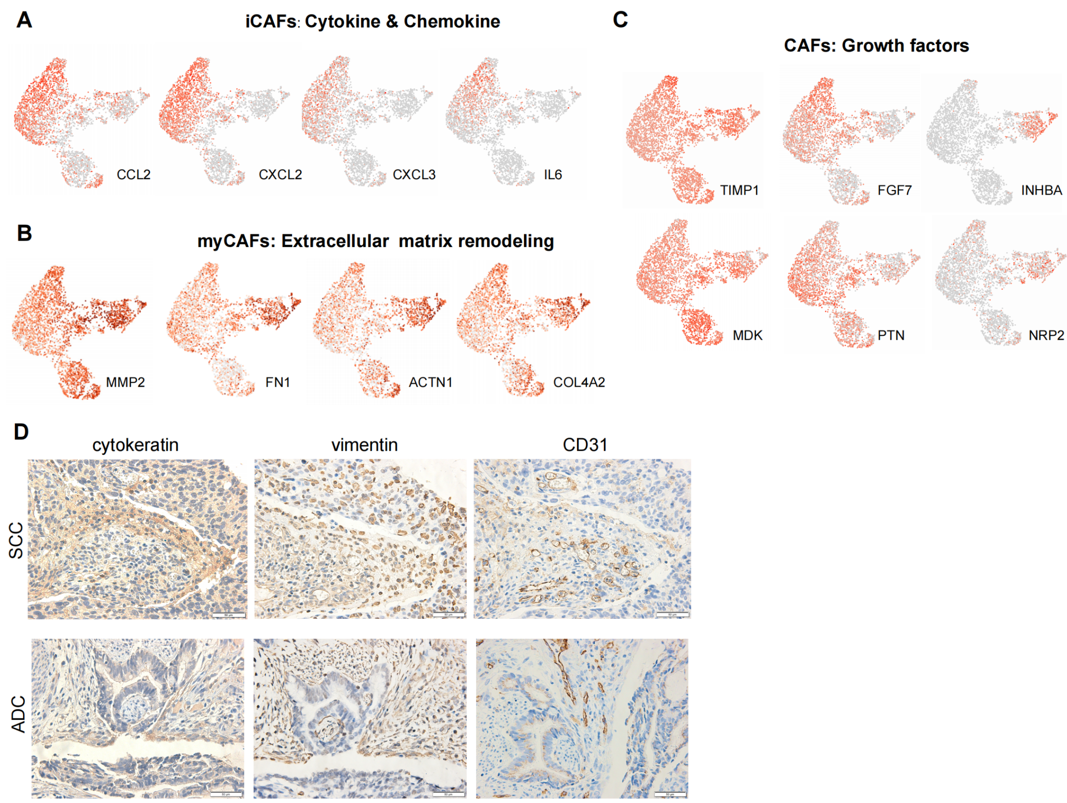

Figure S2. Heterogeneity of CAFs and the distribution of structural cells in CC TME

(A) UMAP projection showing the expression of characteristic cytokines and chemokines (CCL2, CXCL2, CXCL3 and IL6) in iCAFs.

(B) UMAP projection showing the expression of characteristic ECM remodeling-related genes (MMP2, FN1, ACTN1 and COL4A2) in myCAFs.

(C)UMAP projection of growth factors expression in two types of CAFs: TIMP1 and MDK were widely expressed in both iCAFs and myCAFs; FGF7 and PTN were mainly expressed in iCAFs while INHBA and NRP2 were expressed in myCAFs.

(D) IHC staining of CD31 for endothelial cells, cytokeratin for epithelial cells and vimentin for fibroblasts in one SCC sample and one ADC sample. Scale bars, 50um.

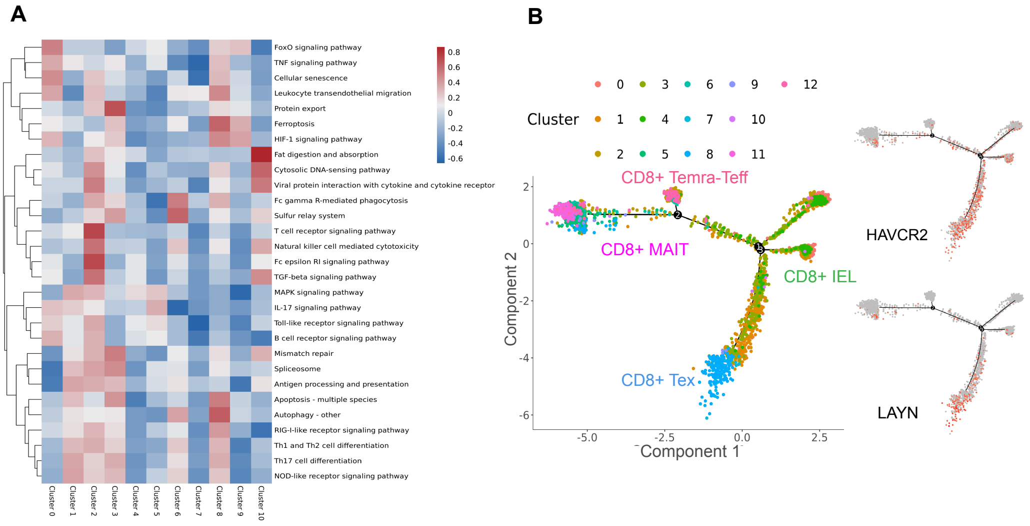

Figure S3. Characteristics and function of T cells in CC TIME

(A)Heatmap of the enriched pathways of each CD4+ T cell cluster through QuSAGE analysis with the color key indicating the enrichment score.

(B)Trajectory of differentiation of CD8+ T cells predicted by monocle with CD8+ Tex cells featuring high expression of HAVCR2 and LAYN, lying at the end point of the trajectory route.

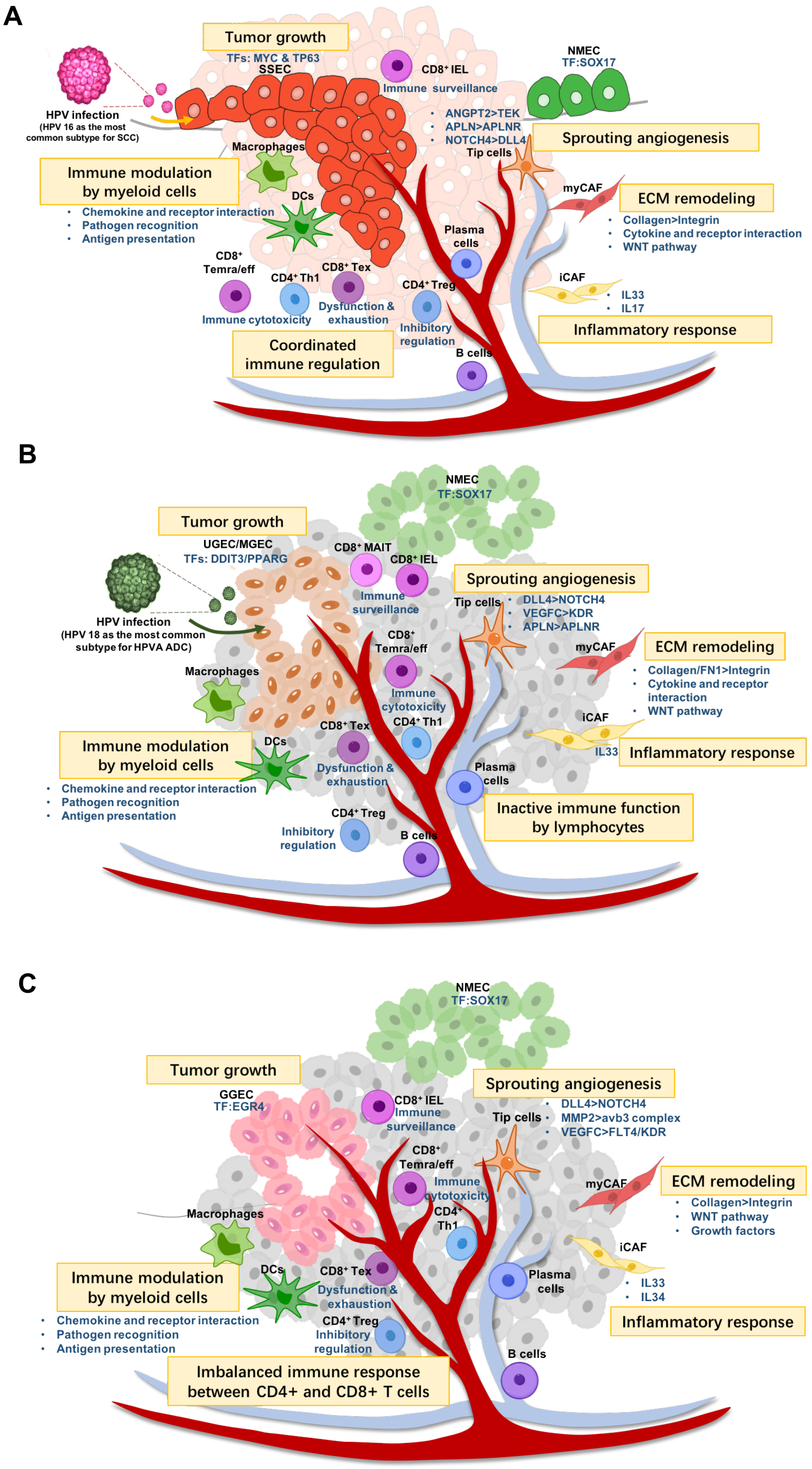

Figure S4. Schematic diagrams of the TME in HPV-positive SCC (A), HPVA ADC (B) and NHPVA ADC (C)

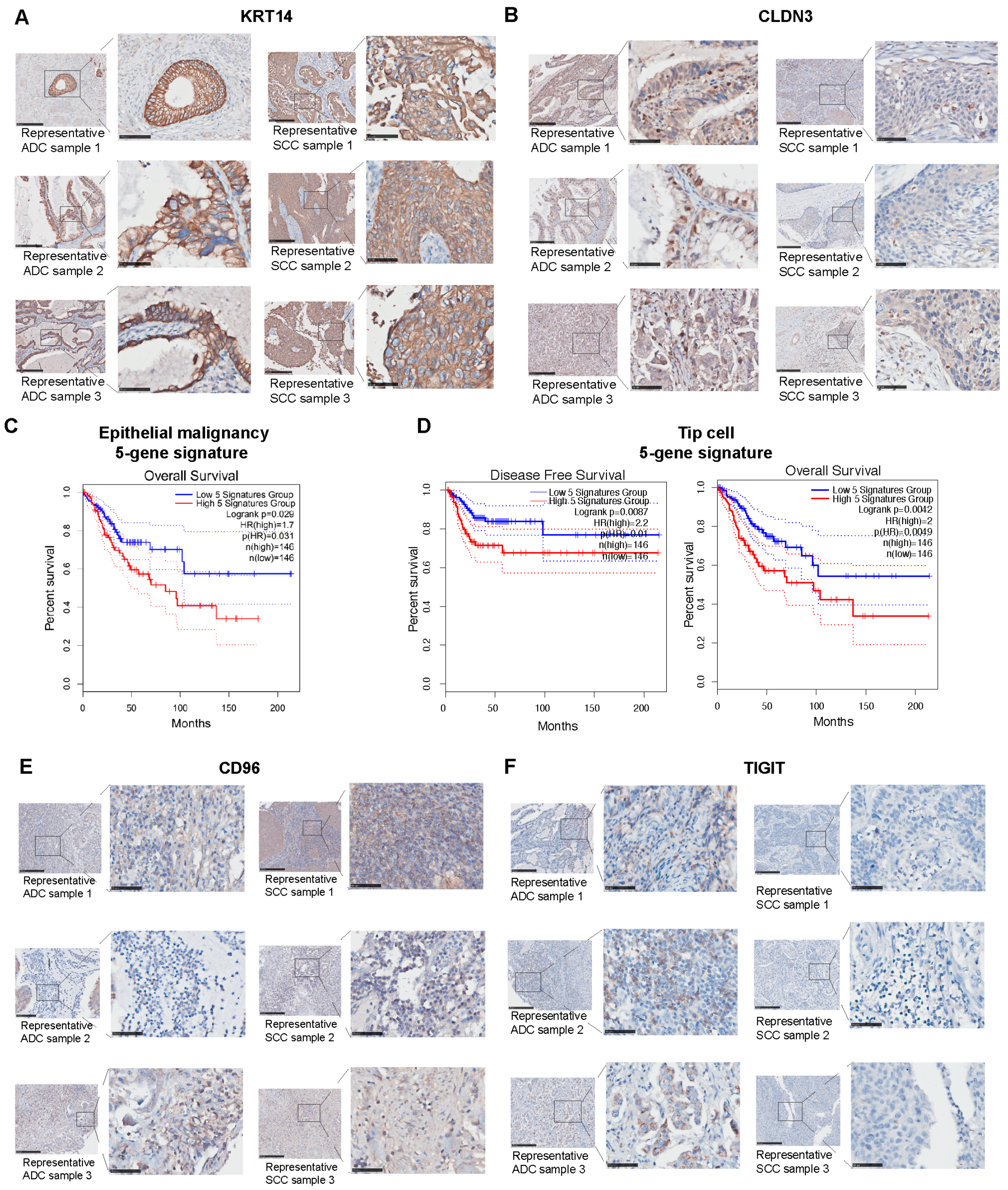

Figure S5. Novel diagnostic and prognostic biomarkers and potential treatment targets for CC

(A) KRT14 expression in three representative ADC samples (scale bars, 250μm and 100/50μm) and three representative SCC samples (scale bars, 250μm and 50μm) by IHC staining.

(B) CLDN3 expression in three representative ADC samples (scale bars, 250μm and 100/50μm) and 3 representative SCC samples (scale bars, 250μm and 50μm) by IHC staining.

(C) Kaplan–Meier curve illustrating the prognostic value of the five-gene malignant epithelial signature (KRT17, S100A10, CDKN2A, CLIC1, KRT20) constructed by the web tool Gepia2 with the CESC TCGA dataset: the higher the signature expression was, the shorter the OS was (p=0.029 by log rank test).

(D) Kaplan–Meier curves illustrating the prognostic value of the five-gene tip cell signature (ESM1, ADAMTSL2, COLA1, APLN, ANGPTL2) constructed by the web tool Gepia2 with the CESC TCGA dataset: the higher the signature expression was, the shorter the OS and DFS was (p=0.0087 and 0.0042 respectively by log rank test).

(E) CD96 expression in three representative ADC samples (scale bars, 250μm and 50μm) and three

representative SCC samples (scale bars, 250μm and 50μm) by IHC staining.
(F) TIGIT expression in three representative ADC samples (scale bars, 250μm and 50μm) and three representative SCC samples (scale bars, 250μm and 50μm) by IHC staining.
